# SENP3 inhibition suppresses hepatocellular carcinoma progression and improves the efficacy of anti-PD-1 immunotherapy

**DOI:** 10.1038/s41418-024-01437-9

**Published:** 2025-01-04

**Authors:** Peng Wang, Jiannan Qiu, Yuan Fang, Songmao Li, Kua Liu, Yin Cao, Guang Zhang, Zhongxia Wang, Xiaosong Gu, Junhua Wu, Chunping Jiang

**Affiliations:** 1 Division of Hepatobiliary and Transplantation Surgery, Department of General Surgery, Nanjing Drum Tower Hospital, the Affiliated Hospital of Medical School, Nanjing University, Nanjing, China; 2 Jinan Microecological Biomedicine Shandong Laboratory, Jinan, China; 3 State Key Laboratory of Pharmaceutical Biotechnology, National Institute of Healthcare Data Science at Nanjing University, Jiangsu Key Laboratory of Molecular Medicine, Medical School, Nanjing University, Nanjing, China; 4 Department of Oncology, the First Affiliated Hospital of Nanjing Medical University, Nanjing, China; 5 Department of Immunology, Key Laboratory of Immune Microenvironment and Disease,, Nanjing Medical University, Nanjing, China; 6 Department of Hepatobiliary and Pancreatic Surgery, The Second Affiliated Hospital of Fujian Medical University, Quanzhou, China

**Keywords:** Oncogenes, Immune evasion

## Abstract

The importance of SUMOylation in tumorigenesis has received increasing attention, and research on therapeutic agents targeting this pathway has progressed. However, the potential function of SUMOylation during hepatocellular carcinoma (HCC) progression and the underlying molecular mechanisms remain unclear. Here, we identified that SUMO-Specific Peptidase 3 (SENP3) was upregulated in HCC tissues and correlated with a poor prognosis. Multiple functional experiments demonstrated that SENP3 promotes the malignant phenotype of HCC cells. Mechanistically, SENP3 deSUMOylates RACK1 and subsequently increases its stability and interaction with PKCβII, thereby promoting eIF4E phosphorylation and translation of oncogenes, including Bcl2, Snail and Cyclin D1. Additionally, tumor-intrinsic SENP3 promotes the infiltration of tumor-associated macrophages (TAMs) while reducing cytotoxic T cells to facilitate immune evasion. Mechanistically, SENP3 promotes translation of CCL20 via the RACK1 /eIF4E axis. Liver-specific knockdown of SENP3 significantly inhibits liver tumorigenesis in a chemically induced HCC model. SENP3 inhibition enhances the therapeutic efficacy of PD-1 blockade in an HCC mouse model. Collectively, SENP3 plays cell-intrinsic and cell-extrinsic roles in HCC progression and immune evasion by modulating oncogene and cytokine translation. Targeting SENP3 is a novel therapeutic target for boosting HCC responsiveness to immunotherapy.

## Introduction

Hepatocellular carcinoma (HCC) is the most prevalent type of liver cancer, accounting for over 90% of primary liver cancers and is one of the leading causes of cancer-related death worldwide [1]. Due to inconspicuous early clinical symptoms, most patients present with advanced HCC at diagnosis and require systemic therapies [2]. Immune checkpoint blockade and tyrosine kinase inhibitors are now well-accepted therapies for the conservative treatment of HCC. However, therapeutic responsiveness to such treatments varies considerably among HCC patients [3]. Therefore, a comprehensive understanding of the molecular mechanisms of HCC development are of paramount importance.

SUMOylation is a common post-translational modification (PTM) in eukaryotic cells that is implicated in many diseases through its involvement in a variety of biological processes [4]. In the presence of SAE, Ubc9 and E3 ligases, substrate proteins undergo a reversible binding process to small ubiquitin-like modifier (SUMO), known as SUMOylation. This process may alter the proteins’ enzymatic activity, cellular sublocalization, stability and the ability to bind other proteins [5]. The SUMO-specific peptidase (SENP) is capable of specifically separating substrate proteins from SUMO binding. In mammalian cells, there are six SENP proteins (SENP1-3 and SENP5-7) with different cellular sublocalization patterns and structures, suggesting that they have distinct functions [6].

In our study, we highlight SENP3 as a potential regulator for HCC because of its high correlation with poor prognosis. We further investigated the effects of SENP3 on HCC cell malignant behaviors and assessed its impact on antitumor immunity. Furthermore, we identified RACK1 as a new target of SENP3 through mass spectrometric analysis. Additionally, we explored the mechanism of tumor-intrinsic SENP3-mediated immune escape using cytokine microarray. Finally, we evaluated the therapeutic effect of SENP3 inhibition in chemically induced HCC and its synergistic effect with anti-PD-1 therapy.

## Results

### SENP3 is highly expressed in HCC and is correlated with a poor prognosis

**Fig. 1:**
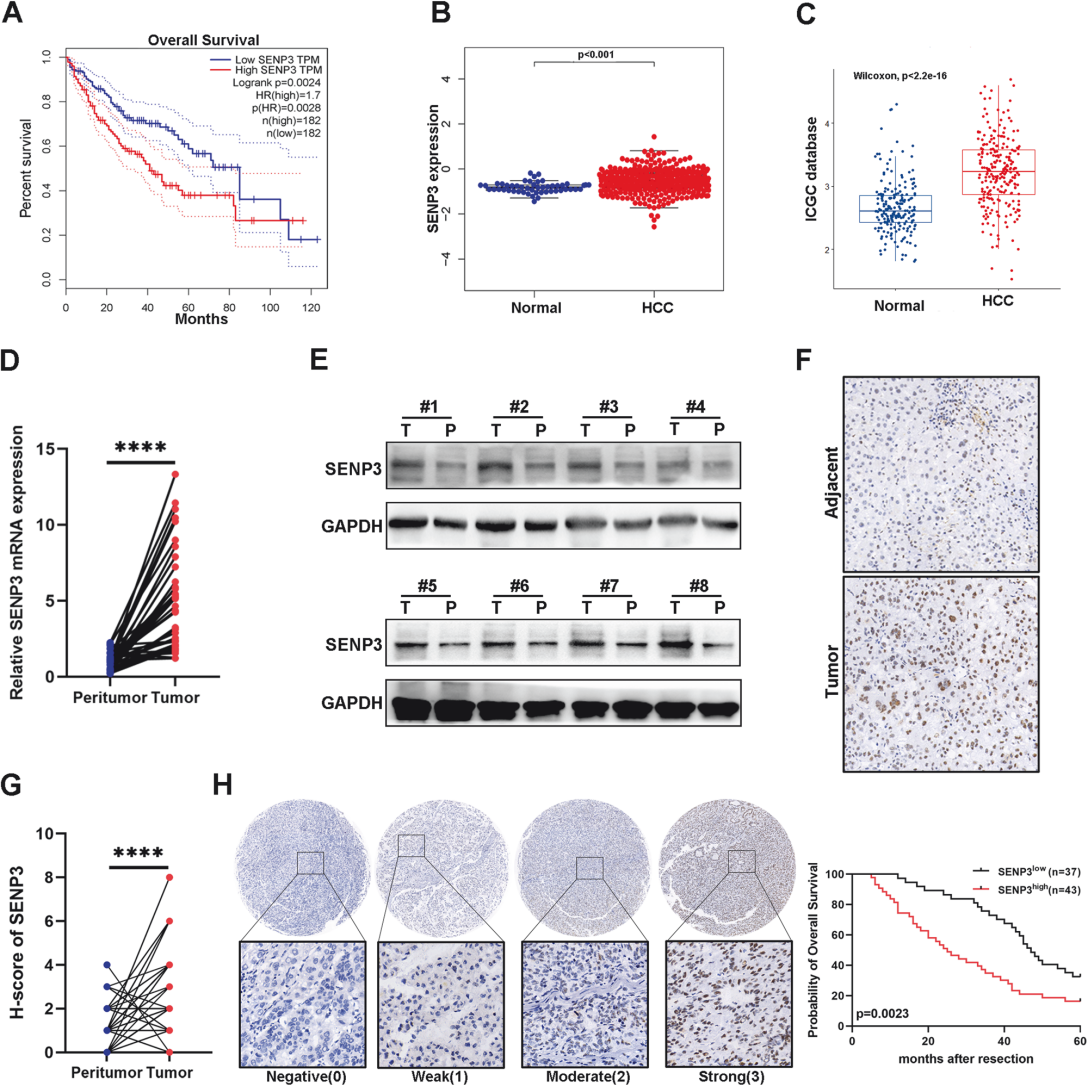
SENP3 is highly expressed in HCC and is correlated with a poor prognosis. **A** Kaplan‒Meier overall survival analysis of HCC patients with different SENP3 expression levels based on the GEPIA database. **B**, **C** SENP3 expression in human HCC tissues and nontumor liver tissues in the TCGA database and the ICGC database. **D** The mRNA expression level of SENP3 in HCC tumor tissues and adjacent nontumor tissues (*n* = 50). **E** The protein expression level of SENP3 in HCC tumor tissues and adjacent nontumor tissues (*n* = 8). **F**, **G** Representative IHC images of SENP3 in HCC tissues and adjacent normal liver tissues and the corresponding H-scores (*n* = 80, Magnification: ×200). **H** Representative IHC images of SENP3 in HCC tumor tissue and the corresponding Kaplan–Meier overall survival (OS) analysis of HCC patients with different SENP3 IHC scores (Magnification: ×200). Two-tailed unpaired Student’s *t* test and log-rank test were performed to determine significance. *P < 0.05, **P < 0.01, ***P < 0.001, ****P < 0.0001.

To screen potential regulators of the SENP family in HCC, overall survival (OS) analysis was performed using the GEPIA database. The results showed that patients with higher levels of SENP1, SENP3 and SENP7 had shorter OS. Among these, the log-rank *p* value for SENP3 in OS analysis was the lowest, but the average mRNA level of SENP3 in multiple HCC cell lines was the highest (Figs. 1A, and S1A, B).

Based on TCGA and ICGC data, SENP3 expression was higher in HCC tissue than in normal liver tissue (Fig. 1B, C). Additionally, elevated SENP3 mRNA and protein levels were observed in our collected HCC tissue samples compared to nontumor tissue samples (Fig. 1D, E). Tissue microarray analysis showed that the HCC tumor tissues had higher SENP3 immunohistochemical (IHC) staining scores (Fig. 1F, G). Besides, we also found that the SENP3-high group had a shorter median OS time (Fig. 1H). SENP3 expression was positively correlated with tumor size, microvascular invasion, TNM stage and Edmonson stage (Table S4). Collectively, these data demonstrate that SENP3 is significantly upregulated in HCC and may be a potential prognostic biomarker for HCC patients.

### SENP3 promotes HCC cell malignant phenotypes in vitro

**Fig. 2:**
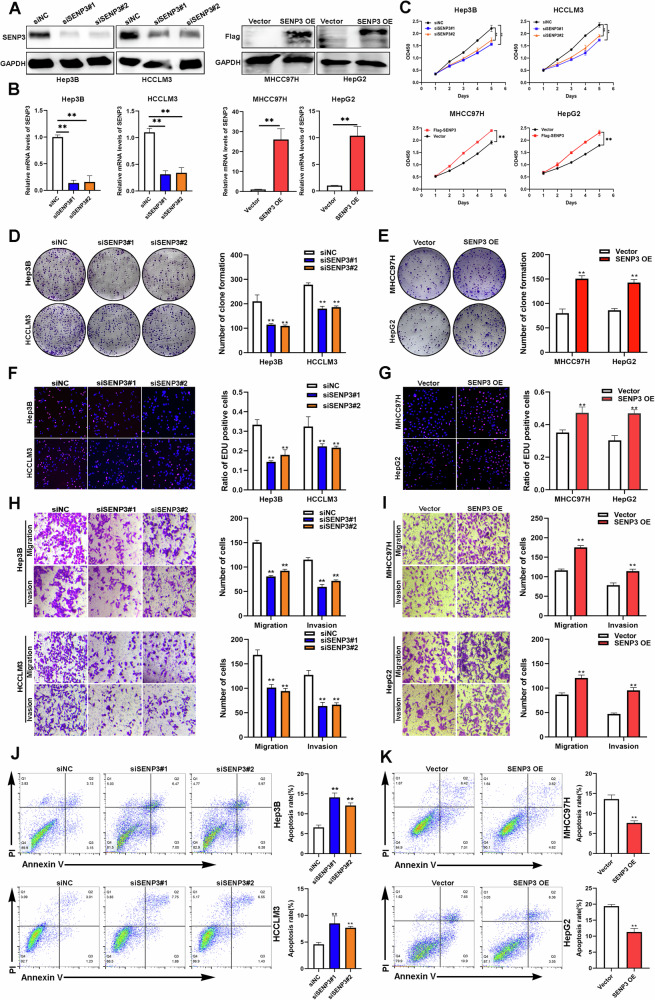
SENP3 promotes HCC cell malignant phenotypes in vitro. **A** The SENP3 knockdown efficiency or overexpression efficiency was confirmed by Western blotting (*n* = 3). **B** The SENP3 knockdown efficiency or overexpression efficiency was confirmed by RT‒PCR analysis (*n* = 3). **C**–**G** The proliferation of Hep3B, HepG2, HCCLM3 and MHCC97H under the influence of SENP3 was evaluated by CCK8 assay, colony formation assay and Edu assay (*n* = 3). **H**, **I** The migratory and invasive capacity of HCC cells with SENP3 overexpression or knockdown were evaluated by using a Transwell assay (*n* = 3). **J**, **K** The apoptosis of HCC cells with SENP3 overexpression or knockdown was evaluated by flow cytometry using the Annexin V FITC/PI staining method (*n* = 3). Two-tailed unpaired Student’s *t* tests were used to determine significance. *P < 0.05, **P < 0.01.

To investigate the influence of SENP3 on cancer hallmarks, including proliferation, invasion and resistance to apoptosis, we conducted a number of in vitro functional experiments. We selected Hep3B, HCCLM3, HepG2 and MHCC97H cells for further studies on the basis of the SENP3 protein level in common HCC cells (Fig. S1C). We then constructed SENP3-knockdown and SENP3-overexpressing cell lines (Fig. 2A, B). The results of the CCK-8, EdU and colony formation assays indicated that SENP3 knockdown inhibited the proliferation of HCC cells, while SENP3 overexpression promoted HCC cell proliferation (Fig. 2C–G). Transwell assays demonstrated that the knockdown and overexpression of SENP3 reduced and increased HCC cell migration and invasion, respectively (Fig. 2H, I). Furthermore, we found that SENP3 knockdown increased the proportion of apoptotic cells, while SENP3 overexpression prevented HCC cell apoptosis (Fig. 2J, K). Moreover, SENP3-deficient HCC cells were generated and confirmed by Western Blot (Fig. S6A), SENP3 deletion inhibited the proliferation and invasion abilities of HCC cells, while promoting the apoptosis of HCC cells (Fig. S6B–E). SENP3 deficiency decreased the snail, Vimentin, and N-cadherin expression, and increased E-cadherin expression (Fig. S6F). As the role of SUMOylation in cellular metabolism and SENP3 serving as a redox sensor have been documented [7, 8], this prompted us to evaluate the effect of SENP3 on cell metabolic properties. A flow cytometer assay demonstrated that SENP3 deletion increased both intracellular and mitochondrial ROS levels (Fig. S7A, B). Furthermore, deletion of SENP3 resulted in relatively higher ATP production (Fig. S7C). Consistently, we found that SENP3 deletion decreased cell glycolysis and increased oxidative phosphorylation (OXPHOS) by using Seahorse XF bioenergetic system (Fig. S7D–G). In summary, these findings demonstrate the tumor-promoting effect of SENP3 on HCC cells in vitro.

### SENP3 promotes HCC growth and metastasis in vivo

**Fig. 3:**
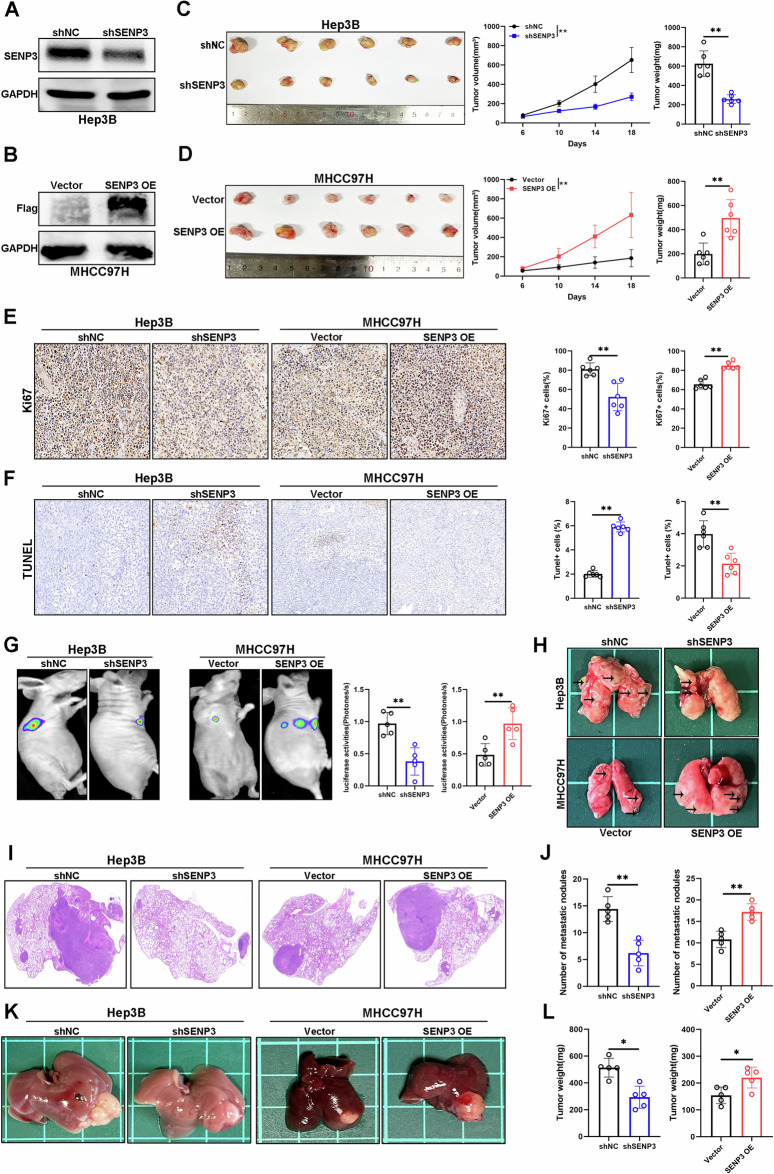
SENP3 promotes HCC growth and metastasis in vivo. **A**, **B** The efficiency of SENP3 knockdown and overexpression in Hep3B and MHCC97H cells was evaluated by Western blotting. **C**, **D** Representative images of nude mouse HCC xenograft tumors in different groups. The tumor volumes and tumor weights are shown in the right panel (*n* = 6). **E** Representative IHC images of Ki67 staining in nude mouse HCC xenograft tumor sections. The relative quantification of positive cells is shown in the right panel (*n* = 6, Magnification: ×200). **F** Representative images of TUNEL staining of nude mouse HCC xenograft tumor sections. The relative quantification of positive cells is shown in the right panel (*n* = 6, Magnification: ×200). **G** Representative bioluminescence images of nude mice after tail vein injection of luciferase-labeled Hep3B and MHCC97H cells. Relative luciferase activity is shown in the right panel (*n* = 5). **H** Representative images of lungs from a nude mouse model of lung metastasis (*n* = 5). **I** Representative images of HE staining of lung tissue from a lung metastasis model (*n* = 5, Magnification: ×20). **J** The number of metastatic foci in lung tissues (*n* = 5). **K**, **L** Representative images of livers with tumors from the orthotopic xenograft liver tumor model and statistical analysis of the tumor weights in different groups (*n* = 5). Two-tailed unpaired Student’s *t* tests were used to determine significance. *P < 0.05, **P < 0.01.

To evaluate the effect of SENP3 on HCC tumor growth and metastasis in vivo, subcutaneous xenograft, lung metastasis, and liver orthotopic xenograft tumor models were established. We constructed stable SENP3-knockdown or SENP3-overexpressing cell lines and confirmed by Western Blot (Fig. 3A, B). In the subcutaneous tumor model, we observed that SENP3 knockdown reduced tumor volume and weight, and SENP3 overexpression exerted the opposite effect on tumor growth (Fig. 3C, D). Consistent with the results of in vitro functional assays, SENP3 knockdown attenuated proliferation and promoted apoptosis in vivo, while the opposite result was observed in the SENP3 overexpression group (Fig. 3E, F). In the metastasis model, the luciferase activity and number of metastases were lower in the SENP3 knockdown group, but SENP3 overexpression promoted the formation of lung metastases (Fig. 3G–J). In the liver orthotopic xenograft tumor model, SENP3 overexpression and knockdown promoted and inhibited the growth of intrahepatic HCC tumors, respectively (Fig. 3K, L). In addition, SENP3 deletion reduced tumor growth in two HCC models (Fig. S6G). Taken together, our findings in different mouse models suggest that SENP3 promotes HCC tumorigenesis and metastasis in vivo.

### SENP3 deconjugates SUMO2/3 from RACK1

**Fig. 4:**
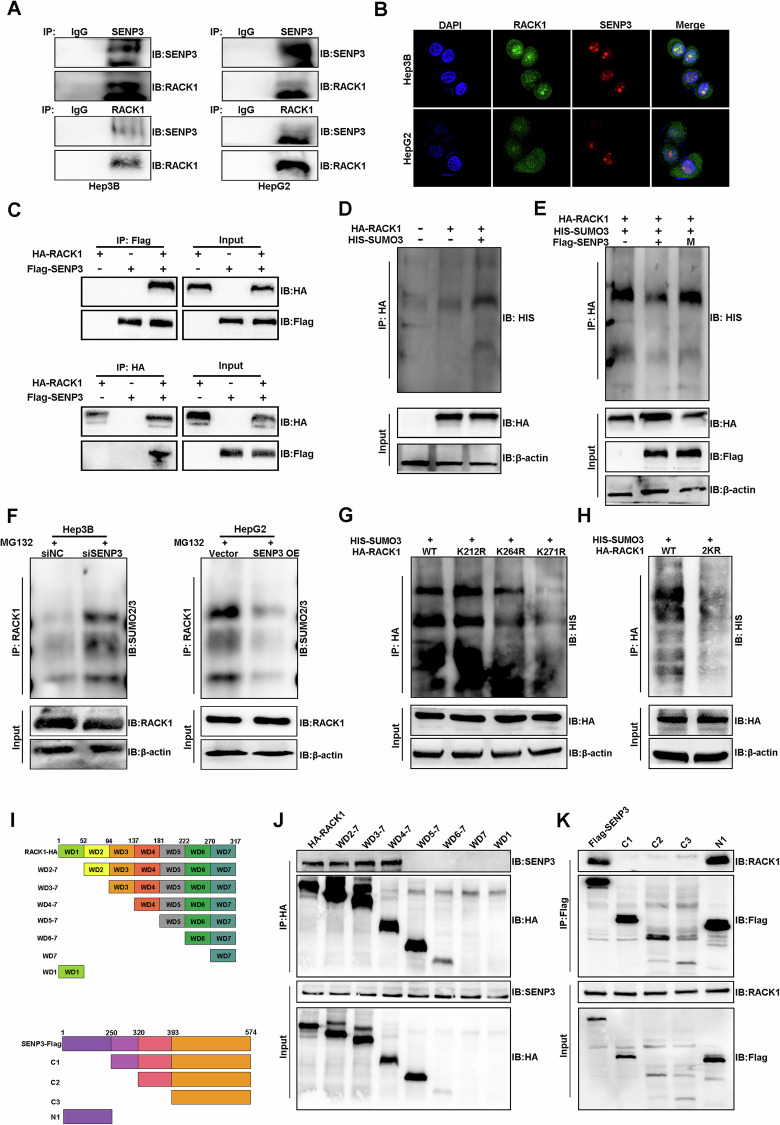
SENP3 deconjugates SUMO2/3 from RACK1. **A** Co-IP and Western blot analyses were performed to confirm the interaction between SENP3 and RACK1 in Hep3B and HepG2 cells. **B** Immunofluorescence assay showing the colocalization of SENP3 with RACK1 in Hep3B and HepG2 cells. **C** Co-IP and Western blot analyses were performed to confirm the exogenous binding between SENP3 and RACK in HEK293T cells transfected with Flag-tagged SENP3 and HA-tagged RACK1. **D** HEK293T cells were transfected with HA-RACK1 and HIS-SUMO3 for 48 h. RACK1 SUMOylation was determined by co-IP and Western blot assays. **E** HEK293T cells were transfected with HA-RACK1, His-SUMO3, and Flag-SENP3 or Flag-SENP3 (C532S). The SUMOylation of HA-RACK1 was determined by co-IP and Western blot assays. **F** Co-IP and Western blot assays were performed to determine RACK1 SUMOylation in Hep3B and HepG2 cells after SENP3 knockdown or overexpression. **G** HEK293T cells were transfected with HIS-SUMO3, HA-tagged wild-type RACK1 or HA-tagged single-site mutant RACK1. SUMOylation assays were performed using HA antibodies. **H** HEK293T cells were transfected with HIS-SUMO3, HA-tagged wild-type RACK1 or HA-tagged double-mutant RACK1. SUMOylation assays were performed using HA antibodies. **I** Schematic diagram of the RACK1 and SENP3 truncations. **J** Co-IP and WB analyses showing the interaction of SENP3 and truncated RACK1 tagged with HA in HEK293T cells transfected with the indicated expression vectors. **K** Co-IP and WB analyses showing the interaction of RACK1 and truncated SENP3 tagged with Flag in HEK293T cells transfected with the indicated expression vectors. All assays were performed in triplicate, and the results of representative experiments are shown.

To investigate the tumorigenic mechanism of SENP3 in HCC, we captured Flag-SENP3 and its binding proteins from transfected HepG2 cells using a co-immunoprecipitation (Co-IP) assay. Subsequent proteomic analysis was performed, and 111 unique proteins were identified. Among all potential SENP3-interacting proteins, the receptor for activated C-kinase 1 (RACK1) had the highest protein score (Fig. S2A, B). Considering that RACK1 is a key scaffolding protein for HCC growth and chemoresistance [9], we hypothesized that RACK1 is a candidate protein regulated by SENP3. The endogenous and exogenous binding of SENP3 to RACK1 was confirmed by co-IP and immunofluorescence analysis (Figs. 4A–C, and S5A). Indeed, our immunoblot analysis confirmed that RACK1 binds primarily to SUMO2/3, the wild-type SENP3 could reduce RACK1 SUMOylation, whereas catalytically inactive SENP3 (C532S) did not (Figs. 4D, E, and S5C, D). Decreased RACK1 SUMOylation was dose-dependently associated with increased SENP3 overexpression (Fig. S5E). Besides, we verified that RACK1 SUMOylation is specifically regulated by SENP3 and not by other members of the SENP family (Fig. S5G). Consistent with these results, the SUMOylation of endogenous RACK1 increased after SENP3 knockdown, but the overexpression of SENP3 reduced the SUMOylation of RACK1 in HCC cells (Figs. 4F, and S5C). Next, the K212, K264 and K271 residues of RACK1 were predicted to be the probable SUMO sites by using SUMO plot and JASSA software. Because of the high similarity between SUMO2 and SUMO3, we chose to study only SUMO3 [8]. The plasmids encoding mutant RACK1 were synthesized and transfected into HEK293T cells. The results demonstrated that RACK1 was conjugated to SUMO3 at residues K264 and K271 (Fig. 4G, H).

To determine which region of RACK1 was responsible for its interaction with SENP3, we constructed seven truncated RACK1 proteins with an HA tag and four truncated SENP3 proteins with a Flag tag based on previous studies [10, 11] (Fig. 4I). The results revealed that SENP3 failed to bind to truncated RACK without amino acids 137-181. The amino acid 1-250 region of SENP3 could interact with full-length RACK1 in HEK293T cells (Fig. 4J, K). Collectively, our findings indicate that SENP3 can deconjugate SUMO3 from RACK1 and that residues K264 and K271 of RACK1 are the main SUMO3 conjugation sites.

### SENP3 increases the stability of RACK1 and promotes its interaction with PKCβII

**Fig. 5:**
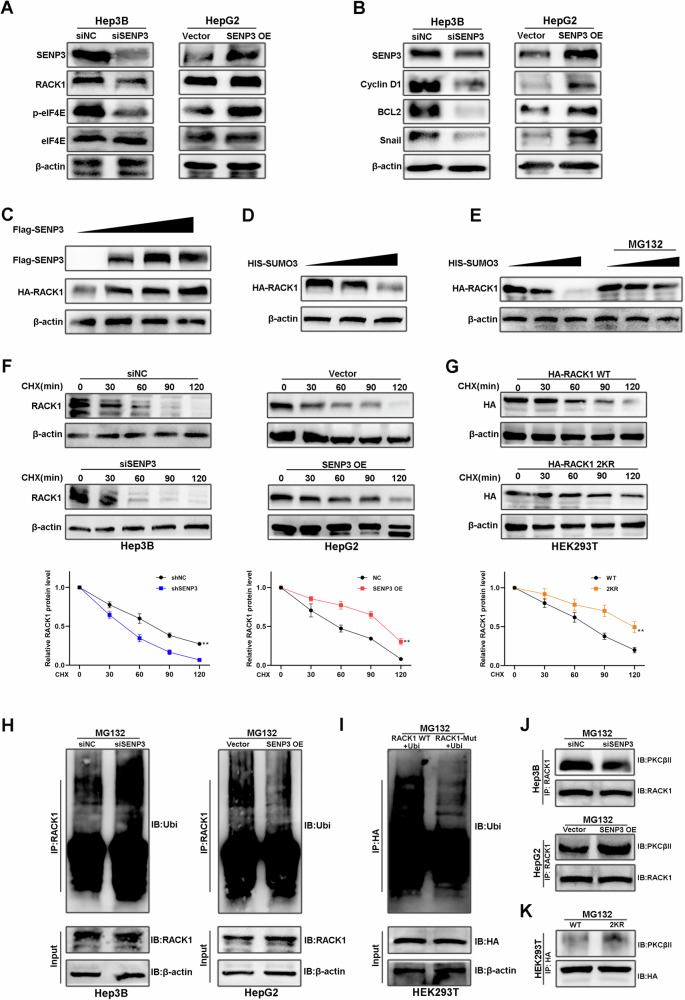
SENP3 promotes the stability of RACK1 and its interaction with PKCβII. **A** The protein levels of RACK1, p-eIF4E, and eIF4E were evaluated by Western blotting in HCC cell lines after knockdown or overexpression of SENP3 in Hep3B and HepG2 cells. **B** The protein levels of BCL2, Cyclin D1 and Snail were evaluated by Western blotting in Hep3B and HepG2 cells after SENP3 knockdown or overexpression. **C** HEK293T cells were transfected with HA-RACK1 and increasing amounts of Flag-SENP3 for 48 h. The protein levels of HA-RACK1 were evaluated by Western blot analysis. **D** HEK293T cells were transfected with HA-RACK1 and increasing amounts of HIS-SUMO3 for 48 h. The protein levels of HA-RACK1 were evaluated by Western blot analysis. **E** HEK293T cells were transfected with HA-RACK1 and increasing amounts of HIS-SUMO3, and the cells were treated with or without MG132 (10 µM) for 10 h. The protein levels of HA-RACK1 were evaluated by Western blot analysis. **F** The protein levels of RACK1 were evaluated by Western blot analysis in SENP3-knockdown or SENP3-overexpressing HCC cells treated with the protein synthesis inhibitor cycloheximide (CHX) for the indicated times. **G** HEK293T cells were transfected with HA-RACK1 or HA-double-mutant RACK1 for 36 h, and then the cells were exposed to CHX for the indicated times. The protein levels of HA-RACK1 were evaluated by Western blot analysis. **H** Ubiquitination assays were performed in SENP3-knockdown or SENP3-overexpressing HCC cells treated with MG132 for 10 h. **I** HEK293T cells were transfected with HA-RACK1 or HA-double-mutant RACK1 and treated with MG132 for 10 h. Whole-cell lysates were collected and used in ubiquitination assays. **J** Co-IP and Western blot analysis were performed to confirm the interaction between RACK1 and PKCβII in HCC cells treated with MG132 for 10 h. **K** HEK293T cells were transfected with HA-RACK1 or HA-double-mutant RACK1 for 36 h and treated with MG132 for 10 h. Co-IP and Western blot analysis were performed to confirm the interaction of PKCβII with wild-type RACK1 or mutant RACK1. All assays were performed in triplicate, and the results of representative experiments are shown.

Growing evidence indicates that the SUMOylation status of a substrate protein affects its stability, subcellular distribution, special function, and ability to bind with other proteins [12]. Thus, we first investigated the effect of SUMOylation on RACK1 stability. We found that SENP3 knockdown suppressed RACK1 expression; the levels of the downstream proteins p-eIF4E, BCL2, Snail and Cyclin D1, which are regulated by RACK1, as previously mentioned, were also reduced after SENP3 knockdown. As expected, the opposite outcome was observed in the SENP3 overexpression group (Figs. 5A, B, and S5B). In addition, the RACK1 protein levels were dose-dependently decreased by HIS-SUMO3 overexpression and increased by SENP3 overexpression, respectively, but the effect of SUMO3 on RACK1 expression was abolished by MG132 treatment (Fig. 5C–E). Interestingly, the RACK1 mRNA levels were not affected by SENP3 expression (Fig. S2C). Therefore, we postulated that SENP3 may regulate RACK1 protein level at posttranslational modification.

In support of this speculation, the half-life of endogenous RACK1 in HCC cells was regulated by SENP3 expression. Moreover, the half-life of the double SUMO site mutant RACK1 (2KR-RACK1) was longer than that of wild-type (WT) RACK1 (Fig. 5F, G). The ubiquitination assays demonstrated that knockdown of SENP3 dramatically increased the polyubiquitination of RACK1, while the autoubiquitination and proteasomal degradation of RACK1 were prevented when SENP3 was overexpressed in HCC cells (Fig. 5H). Compared with that of WT RACK1, the ubiquitination of double-mutant RACK1 was also decreased (Fig. 5I). RACK1 is known to cooperate with PKCβII to promote the phosphorylation of eIF4E [9]. We thus assessed the binding between RACK1 and PKCβII. Our results revealed that the association of these two molecules was impaired in SENP3-knockdown cells (Fig. 5J). Similarly, mutations in the SUMO site promoted the interaction of RACK1 and PKCβII (Fig. 5K). Together, these results suggest that deSUMOylation increases RACK1 stability and promotes its interaction with PKCβII in HCC cells.

### SENP3 promotes HCC malignancy via the RACK1/eIF4E axis

The RACK1/eIF4E axis modulates HCC chemoresistance and growth, which has been well documented, and we observed that a reduction in RACK1 and p-eIF4E expression after SENP3 knockdown, thus we speculated that SENP3 promotes HCC progression through RACK1-dependent modulation of eIF4E. To confirm this hypothesis, rescue experiments were performed by overexpressing wild-type RACK1 or 2KR-RACK1 in SENP3-knockdown HCC cells. As expected, we found that stable overexpression of WT-RACK1 or 2KR-RACK1 rescued the SENP3 knockdown-induced effects on HCC cell malignant behaviors. More importantly, the malignant phenotypes in shSENP3 cells were more profoundly reversed by 2KR-RACK1 than in shSENP3 cells transduced with WT-RACK1 (Fig. S3A–I). To elucidate the critical role of eIF4E in the oncogenic process driven by SENP3, we silenced eIF4E using the corresponding lentivirus as previously reported and then re-overexpressed wild-type eIF4E and the S209A mutant eIF4E [13] (Fig. S4A). Our results demonstrated that SENP3-mediated HCC cell malignancy was partially through eIF4E, besides, S209A eIF4E failed to rescue eIF4E knockdown-induced effect on SENP3 overexpressing HCC cells (Fig. S4B–F). Thus, our data suggest that SENP3 regulates HCC progression and metastasis through the RACK1-EIF4E axis.

### Tumor-intrinsic SENP3 reshapes the immune microenvironment by promoting the infiltration of tumor-associated macrophages in HCC

**Fig. 6:**
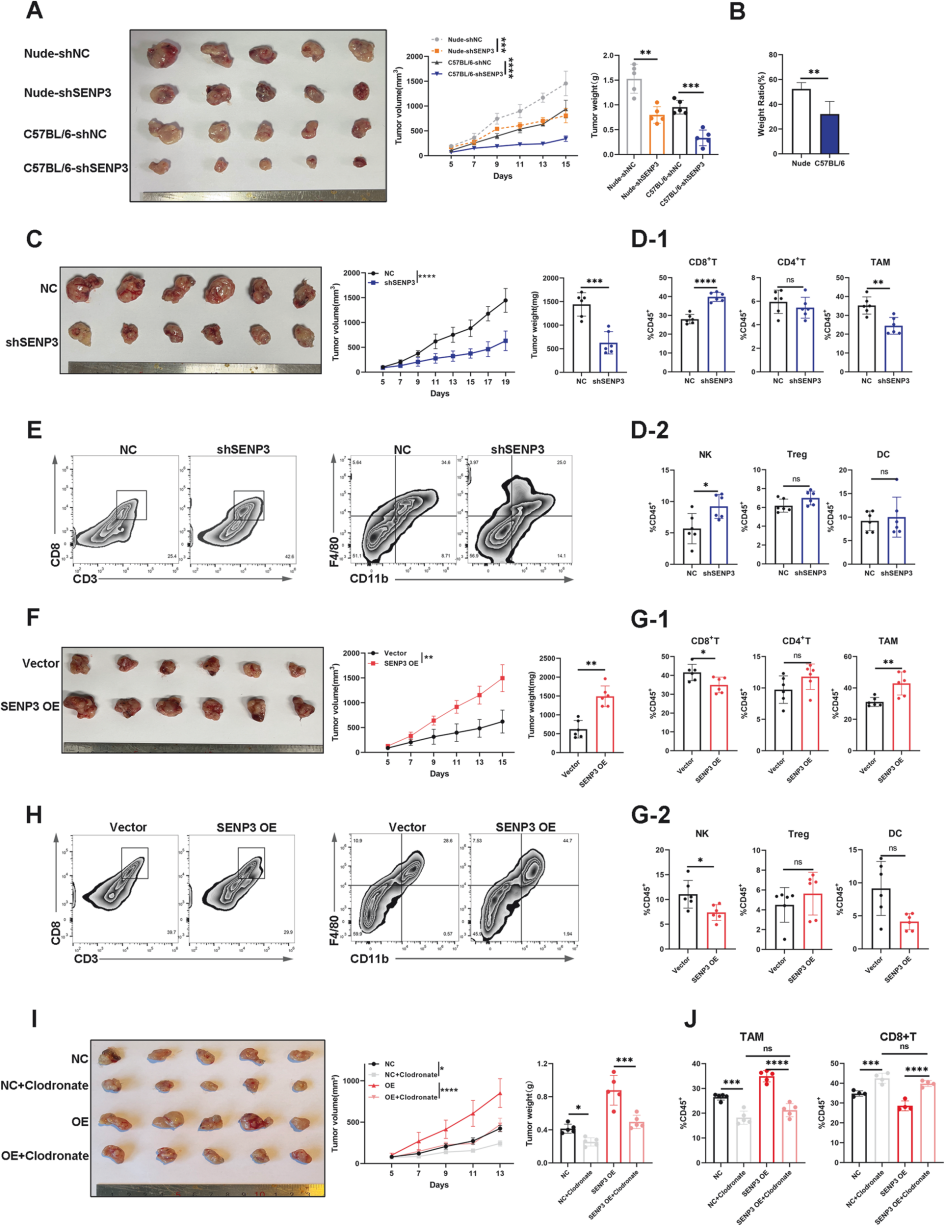
Tumor-intrinsic SENP3 reshapes the immune microenvironment by promoting the infiltration of tumor-associated macrophages in HCC. **A** Representative images of subcutaneous Hepa1-6 tumors in immunocompromised mice (Nude) and immunocompetent (C57BL/6) mice. The tumor volumes and tumor weights are shown in the right panel. **B** Degree of tumor weight reduction after SENP3 knockdown in C57BL/6 mice and Nude mice. Weight ratio=shSENP3(g)/NC(g)×100%. **C** Representative images of subcutaneous Hepa1-6 tumors collected from the NC or shSENP3 groups. The tumor volumes and tumor weights are shown in the right panel (*n* = 6). **D** Statistical analysis of tumor-infiltrating lymphocytes (CD8+ T cells, CD4+ T cells, TAMs, NK cells, DCs and Tregs) from different groups according to the flow cytometry results (*n* = 6). **E** Representative flow cytometric analysis of CD8+ T cells and TAMs in Hepa1-6 tumors from the shSENP3 and NC groups (*n* = 6). **F** Representative images of subcutaneous Hepa1-6 tumors collected from the Vector or SENP3 OE groups. The tumor volumes and tumor weights are shown in the right panel (*n* = 6). **G** Statistical analysis of tumor-infiltrating lymphocytes (CD8+ T cells, CD4+ T cells, TAMs, NK cells, DCs and Tregs) from different groups according to the flow cytometry results (*n* = 6). **H** Representative flow cytometric analysis of CD8+ T cells and TAMs in Hepa1-6 tumors from the Vector or SENP3 OE groups (*n* = 6). **I** Representative images of harvested tumors from different groups. The clodronate liposome was used for macrophage depletion. The tumor volumes and tumor weights are shown in the right panel (*n* = 5). **J** Statistical analysis of CD8+ T cells and TAMs in hepa1-6 tumors from different groups according to the flow cytometry results (*n* = 5). Two-tailed unpaired Student’s *t* test or one-way ANOVA was used to determine significance. *P < 0.05, **P < 0.01, ***P < 0.001, ****P < 0.001.

To study whether SENP3-mediated deSUMOylation affects HCC immune microenvironment, we established subcutaneous tumor models in immunocompetent and immunodeficient mice, respectively. We found that tumor-intrinsic SENP3 deficiency inhibited HCC tumor growth more strongly in C57BL/6 mice than in Nude mice, suggesting that SENP3-induced HCC tumorigenesis is partially mediated by immune function (Figs. 6A, B, and S2D). SENP3 knockdown significantly inhibited tumor growth and prolonged overall survival, the opposite results were observed in SENP3 overexpression group. Importantly, a reduction in tumor-associated macrophages (TAM) infiltration and an increase in CD8 + T cells and NK cells were observed in the SENP3 knockdown group; whereas SENP3 overexpression exerted the opposite effect (Figs. 6C–H, and S2E, F). Overall, these data suggest that SENP3 promotes immune escape in HCC. Given that TAM suppresses CD8+ T-cell proliferation and activation, thereby facilitating HCC progression [14, 15], we hypothesized that SENP3-mediated immunosuppression in HCC may act through TAM. The results showed that deletion of macrophages reversed the SENP3-mediated pro-tumorigenic effect (Fig. 6I). Macrophage deletion abolished the SENP3-mediated alteration in the ratio of CD8 + T cells (Fig. 6J). Besides, the M2 polarization of TAM at the tumor site was not affected regardless of SENP3 knockdown or overexpression (Fig. S2G). Thus, our study confirms that SENP3 creates an immunosuppressive microenvironment via recruiting TAM.

### SENP3 promotes tumor-associated macrophages recruitment by upregulating CCL20 expression

**Fig. 7:**
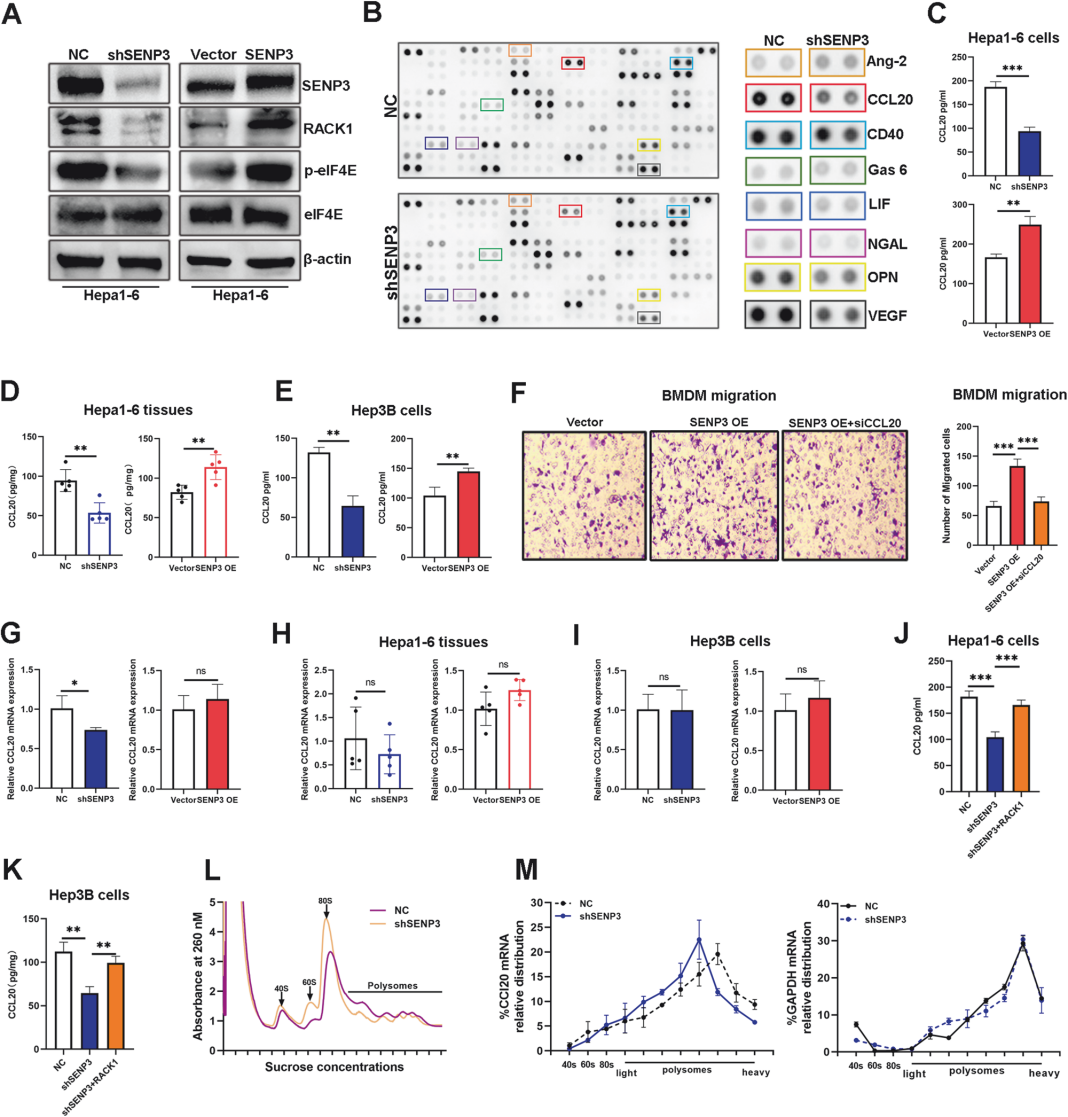
SENP3 promotes tumor-associated macrophages recruitment by upregulating CCL20 expression. **A** The protein levels of RACK1, p-eIF4E, and eIF4E were evaluated by Western blot after SENP3 knockdown or overexpression in Hepa1-6 cells (*n* = 3). **B** Factors secreted by NC and shSENP3 Hepa1-6 cells were detected by a mouse cytokine array, and a representative image of the cytokine array is shown (*n* = 2). **C** Secreted CCL20 protein levels in Hepa1-6 cells after SENP3 knockdown or overexpression were detected by ELISA (*n* = 3). **D** CCL20 protein levels in Hepa1-6 tumors after SENP3 knockdown or overexpression were evaluated by ELISA (*n* = 5). **E** Secreted CCL20 protein levels in Hep3B cells after SENP3 knockdown or overexpression (*n* = 3). **F** The migration ability of BMDMs toward Hepa1-6 conditioned media from different groups was determined by Transwell assays. Representative images of migrated BMDMs are shown (*n* = 3). **G** CCl20 mRNA levels were detected by RT‒PCR in Hepa1‒6 cells after SENP3 knockdown or overexpression (*n* = 3). **H** CCL20 mRNA levels in Hepa1-6 tumors after SENP3 knockdown or overexpression were evaluated by RT-PCR (*n* = 5). **I** CCL20 mRNA levels in Hep3B cells after SENP3 knockdown or overexpression were evaluated by RT-PCR (*n* = 3). **J** CCL20 protein levels in SENP3-knockdown Hepa1-6 cells transfected with RACK1 plasmid were detected by ELISA (*n* = 3). **K** CCL20 protein levels in SENP3-knockdown human HCC cells transfected with RACK1 plasmid were detected by ELISA (*n* = 3). **L**, **M** Polysome profiling of Hepa1-6 cells with or without SENP3 knockdown. CCl20 and GAPDH mRNA expression in different polysome fractions were evaluated by RT‒PCR analysis (*n* = 3). Two-tailed unpaired Student’s *t* test or one-way ANOVA was used to determine significance. *P < 0.05, **P < 0.01, ***P < 0.001.

Given recent reports that phospho-eIF4E promotes immunosuppression in melanoma by regulating cytokine translation [16], we also observed SENP3-RACK1-eIF4E axis was existed in hepa1-6 cells (Fig. 7A). Thus, we hypothesized that SENP3 contributes to immunosuppression by controlling cytokine production. A mouse cytokine array was performed and results showed that CCL20, CD40, Gas6, LIF, NGAL, OPN, and VEGF were decreased, while Ang2 was increased in the SENP3-knockdown group (Fig. 7B). HCC cell-derived CCL20 can directly promote the accumulation of TAMs in the tumor immune microenvironment (TIME), which subsequently leads to a reduction of CD8+ T cells [17]. ELISA assays confirmed that the production of CCL20 from human or murine HCC cells was altered by SENP3 expression (Fig. 7C–E). In addition, SENP3 overexpression promoted the recruitment of bone marrow-derived macrophages (BMDMs) through in vitro chemotaxis assays, and the promotion of BMDM migration induced by SENP3 overexpression was largely inhibited after silencing CCL20 expression (Figs. 7F, and S2H). Intriguingly, SENP3 knockdown slightly downregulated CCL20 mRNA expression and that SENP3 overexpression did not affect CCL20 mRNA expression (Fig. 7G). Consistently, no obvious differences in CCL20 mRNA levels in tumor tissues or in human HCC cells from different groups (Fig. 7H, I). Therefore, we hypothesized that SENP3 promotes CCL20 protein expression mainly by modulating its translation rate.

The rescue experiments showed that overexpressing RACK1 abolished the effect of SENP3 knockdown on CCL20 secretion (Fig. 7J, K). The polysome-profiling results showed that the distribution of CCL20 mRNA had a significant shift from heavy to light polysome fractions in the SENP3 knockdown group compared to that in the control group, while the distribution of GAPDH mRNA in the polysome fraction was not affected by SENP3 knockdown, indicating that the translation efficiency of CCL20 decreased after SENP3 knockdown (Fig. 7L, M). These results suggest that HCC-derived SENP3 promotes macrophage recruitment by enhancing CCL20 translation in a RACK1-dependent manner.

### Targeting SENP3 inhibits the progression of HCC and potentiates the efficacy of anti-PD-1 therapy

**Fig. 8:**
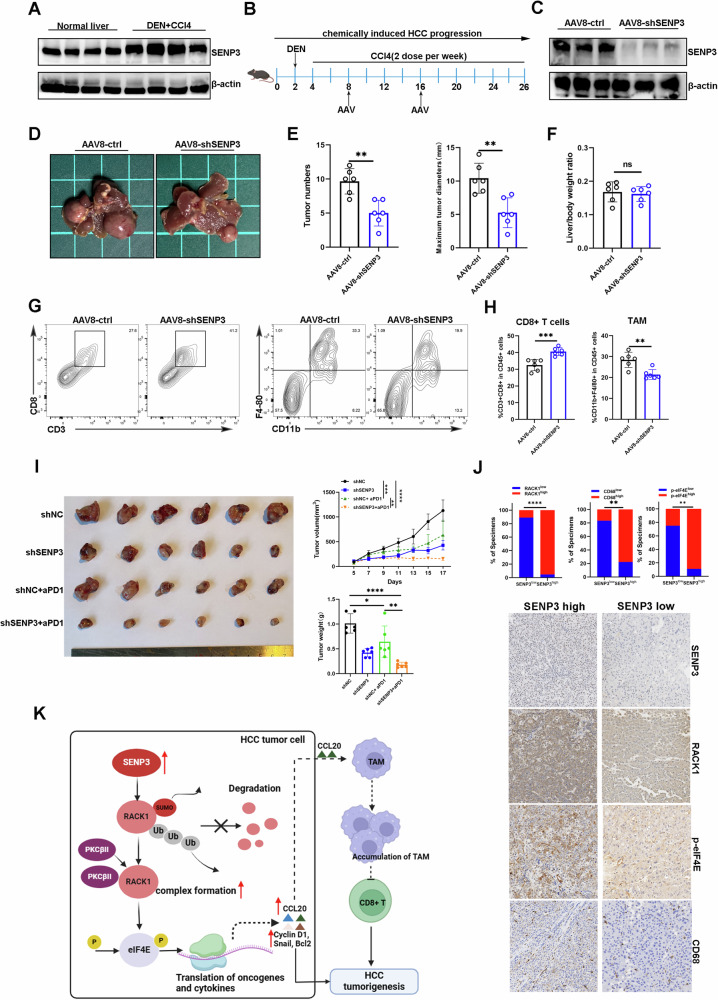
Targeting SENP3 inhibits the progression of HCC and potentiates the efficacy of anti-PD-1 therapy. **A** The protein level of SENP3 in DEN/CCL4-induced HCC tumors were evaluated by Western blotting (*n* = 4). **B** Schematic representation of in vivo experiments to assess the effect of AAV8-shSENP3 treatment on tumor development in DEN/CCL4-induced HCC models. **C** Validation of SENP3 expression in livers after AAV8-shNC or shSENP3 administration using western blotting (*n* = 3). **D** Representative images of livers with tumors from the AAV8-shNC and shSENP3 treatment groups (*n* = 6). **E** Quantification of tumor numbers and maximum tumor sizes in the livers of mice in the AAV8-shNC and shSENP3 treatment groups (*n* = 6). **F** Liver weight/body weight ratio in the indicated groups (*n* = 6). **G** Representative flow cytometric analysis of CD8+ T cells and TAMs in chemically induced tumors from different groups (*n* = 6). **H** Statistical analysis of tumor-infiltrating lymphocytes (CD8+ T cells, TAMs) from different groups according to the flow cytometry results (*n* = 6). **I** Hepa1-6-NC and Hepa1-6-shSENP3 tumor-bearing C57BL/6 mice were treated with anti-PD-1 or IgG when tumors reached 80–130 mm^3^. Representative images of HCC tumors from different groups. Tumor volumes and tumor weights are shown in right panel. **J** Representative IHC staining of serial human tumor tissue sections for SENP3, RACK1, p-eIF4E, CD68 (scale bars, 100 µm) (*n* = 30). And the correlation between SENP3 and RACK1 or SENP3 and p-eIF4E or SENP3 and CD68 was determined by Chi-square test. **K** A schematic diagram showing the mechanism by which SENP3/RACK1 promotes cancer cell malignant behavior and tumor immune evasion. Two-tailed unpaired Student’s *t* test or one-way ANOVA was used to determine significance. *P < 0.05, **P < 0.01, ***P < 0.001, ****P < 0.001.

To further determine whether knocking down SENP3 in the liver might inhibit the progression of HCC, we established a chemically induced HCC model and treated mice with AAV8- shSENP3 and AAV8-ctrl viruses (Fig. 8B). Increased SENP3 expression was observed in DEN-CCl4-induced HCC tissues compared with normal liver tissues (Fig. 8A). We also confirmed the knockdown efficiency of AAV8-shSENP3 in the liver through immunoblot analysis (Fig. 8C). As expected, the analysis of livers from DEN-CCl4-treated mice showed that SENP3 knockdown reduced tumor number and size (Fig. 8D–F), which confirms that targeting SENP3 has therapeutic potential during HCC progression. In addition, more CD8+ T cells and fewer TAMs were observed in HCC tissues from mice treated with AAV8-shSENP3, in comparison to those from the control group (Fig. 8G, H). Given the improvement in TIME after SENP3 knockdown, we speculated that SENP3 deficiency may potentiate anti-PD1 efficacy in HCC. Indeed, we found that anti-PD1 slightly inhibited the tumor growth in Hepa1-6 NC groups, while anti-PD1 combined with SENP3 knockdown significantly suppressed HCC tumor growth (Fig. 8I). Thus, these results suggest that targeting SENP3 enhances the efficacy of anti-PD1 therapy in HCC. To explore the clinical relevance of the above findings, we performed IHC staining on serial HCC tissue sections. We discovered that the SENP3 protein levels were positively correlated with the RACK1 and p-eIF4E protein levels, and we also observed increased expression of CD68 (a marker for TAMs) in the SENP3-high group, which indicates that the SENP3-RACK1-eIF4E axis and an immunosuppressive microenvironment also exist in human HCC samples (Fig. 8J). Immunoblot analysis also demonstrated that RACK1 and phospho-eIF4E were increased, while SUMOylated RACK1 was decreased in SENP3-higher group (Fig. S5F). Collectively, our findings suggest that SENP3 inhibition could be a potential therapeutic approach for treating advanced HCC patients.

## Discussion

Accumulating evidence has suggested that SUMOylation is involved in cancer pathogenesis, and some studies of drugs targeting the SUMO modification process (such as TAK-981 and momordin Ιc) have made great progress [18–20]. Research also shows that SENP-mediated deSUMOylation is critical for the development of some tumors, suggesting that SENPs may be promising therapeutic targets [6]. In our study, we identified SENP3 was upregulated in HCC and strongly associated with poor prognosis in patients with HCC. In agreement with this, a previous study reported the increased SENP3 expression in HCC cells obtained from tissue samples [21]. In addition, we found that SENP3 promotes the proliferation, invasion, and metastasis of HCC cells through various loss- and gain-of-function experiments. Importantly, the antitumor effect of liver-specific SENP3 knockdown in the DEN-CCl4-induced HCC model was validated by AAV8 administration. Overall, our study demonstrates the tumor-promoting role of SENP3 in HCC and that targeting SENP3 is a promising approach for HCC treatment.

Typically, SENP3 exerts its biological effects by regulating the function of substrate proteins through its catalytic activity [22, 23]. RACK1 is a scaffold protein for several kinases and receptors that acts as a component of the 40S ribosome and cooperates with PKCβII to promote the translation of CyclinD1, Snail, BCL2 and Myc, thereby promoting HCC cell proliferation and drug resistance [9, 24]. SENP3 is predominantly located in the nucleolus, SENP3 and SENP5 inactivation disrupts the ribosomal subunit assembly pathway [25]. These reports suggest that the crosstalk between SENP3 and RACK1 may exist and that the SENP3/RACK1 axis may play a role in ribosome function. Here, we first confirmed that SENP3 can bind to RACK1 and regulate the deSUMOylation of RACK1 at residues K264 and K271. In addition, follow-up studies also confirmed that the SENP3 plays a role in ribosome translation partially in a RACK1-dependent manner.

SUMO modifications regulate the activity of substrate proteins by affecting their stability, subcellular localization, and interactions with other functional proteins [26]. In our study, we confirmed that the SUMOylation of RACK1 affects its stability by modulating its ubiquitination-mediated degradation. As described above, RACK1 cooperates with PKCβII to promote the phosphorylation of eIF4E. We therefore investigated the interaction of RACK1 with PKCβII and found increased binding between these two molecules after SENP3 overexpression. Moreover, the levels of downstream molecules (including p-eIF4E, cyclin D1, snail, and Bcl2) regulated by RACK1 were also decreased after SENP3 knockdown, which is consistent with the impact of SENP3 on cancer hallmarks.

Recent research has shown that the endogenous effects of a molecule on tumor cells may not reflect its function in the immune microenvironment [27]. A previous study indicated that eIF4E phosphorylation potentiates antitumor immune responses by promoting CCL5 translation [16]. Another article indicated that the MNK1/2-eIF4E axis supports immune evasion in postpartum breast cancer by regulating IL33 protein levels [28]. Thus, we further analyzed immune cells in HCC tumors from immunocompetent mice after SENP3 knockdown/overexpression. We observed that tumor-intrinsic SENP3 promotes the infiltration of TAMs, while reducing the accumulation of CD8+ T cells, resulting in an immunosuppressive microenvironment. Furthermore, we revealed that the combination of SENP3 knockdown with anti-PD-1 treatment reduced HCC tumor growth more effectively than either anti-PD1 therapy or SENP3 inhibition alone. Our work suggests that targeting SENP3 may be an effective way to overcome HCC resistance to immunotherapy.

Cytokines secreted by tumor cells play a crucial role in regulating the immunosuppressive tumor microenvironment [29, 30]. We observed that SENP3 knockdown resulted in altered expression of a variety of cytokines. Previous studies have shown that CCL20 can directly recruit TAMs to inhibit CD8+ T-cell function and ultimately create an immunosuppressive microenvironment [17, 31, 32]. Therefore, we focused on the process that tumor-intrinsic SENP3 promotes macrophage infiltration by augmenting CCL20 secretion.

Next, we investigated the molecular mechanism of the SENP3-mediated CCL20 upregulation. We observed that SENP3 knockdown/overexpression had a marginal effect on CCL20 mRNA levels in HCC cells. In addition, the SENP3-RACK1-eIF4E axis was also present in Hepa1-6 cells. As previously stated, phosphorylation of eIF4E can improve the translation efficiency of certain cytokines [16]. Another study showed that PKR/EIF2α activation inhibits the translation of CCL20 in macrophages [33], suggesting that the translation efficiency of CCL20 is finely regulated in ribosomes. Hence, we speculated that SENP3 may affect the protein level of CCL20 at the translational level. Consistently, polysome profiling analysis strongly confirmed that SENP3 promoted CCL20 translation in HCC cells. Thus, this study provides the first evidence that SENP3 reshapes the immune microenvironment by enhancing CCL20 translation in a RACK1-dependent manner.

In conclusion, SENP3 drives HCC progression by regulating the malignant phenotypes of HCC cells and the immunosuppressive microenvironment. Mechanistically, SENP3 represses the SUMOylation of RACK1 at K264 and K271. This increases the stability of RACK1 and its ability to synergize with PKCβII, thereby facilitating BCL2, Snail, Cyclin D1 and CCL20 translation. Precise targeting of SENP3 may be an effective and promising option for the clinical treatment of HCC.

## Methods and materials

The detailed methods and materials are described in the supplementary information.

## Supplementary information

Supplementary Figure 1

Supplementary Figure 2

Supplementary Figure 3

Supplementary Figure 4

Supplementary Figure 5

Supplementary Figure 6

Supplementary Figure 7

Supplementary Figure 8

Supplementary Information

Uncropped original western blots

## Data Availability

The datasets used in the analysis can be obtained by contacting the corresponding author on reasonable request.
