# Correction: SENP3 inhibition suppresses hepatocellular carcinoma progression and improves the efficacy of anti-PD-1 immunotherapy

**DOI:** 10.1038/s41418-025-01455-1

**Published:** 2025-02-06

**Authors:** Peng Wang, Jiannan Qiu, Yuan Fang, Songmao Li, Kua Liu, Yin Cao, Guang Zhang, Zhongxia Wang, Xiaosong Gu, Junhua Wu, Chunping Jiang

**Affiliations:** 1 Division of Hepatobiliary and Transplantation Surgery, Department of General Surgery, Nanjing Drum Tower Hospital, the Affiliated Hospital of Medical School, Nanjing University, Nanjing, China; 2 Jinan Microecological Biomedicine Shandong Laboratory, Jinan, China; 3 State Key Laboratory of Pharmaceutical Biotechnology, National Institute of Healthcare Data Science at Nanjing University, Jiangsu Key Laboratory of Molecular Medicine, Medical School, Nanjing University, Nanjing, China; 4 Department of Oncology, the First Affiliated Hospital of Nanjing Medical University, Nanjing, China; 5 Department of Immunology, Key Laboratory of Immune Microenvironment and Disease, Nanjing Medical University, Nanjing, China; 6 Department of Hepatobiliary and Pancreatic Surgery, The Second Affiliated Hospital of Fujian Medical University, Quanzhou, China

**Keywords:** Oncogenes, Immune evasion

Correction to: *Cell Death & Differentiation* 10.1038/s41418-024-01437-9, published online 04 January 2025

In the initial published version of this article, there was an error in the author information. Zhongxia Wang (freud_t@126.com); Xiaosong Gu(nervegu@ntu.edu.cn) and Junhua Wu(wujunhua@nju.edu.cn) are also co-corresponding authors. This correction does not affect the description of the results or the conclusions of this work.

The original article has been corrected.

