# Supplementary figures and images for: SENP3 inhibition suppresses hepatocellular carcinoma progression and improves the efficacy of anti-PD-1 immunotherapy
Source: Cell Death Differ. 2025 Jan 4;32(5):959–72. doi: 10.1038/s41418-024-01437-9 (PMC12089275; doi:10.1038/s41418-024-01437-9)

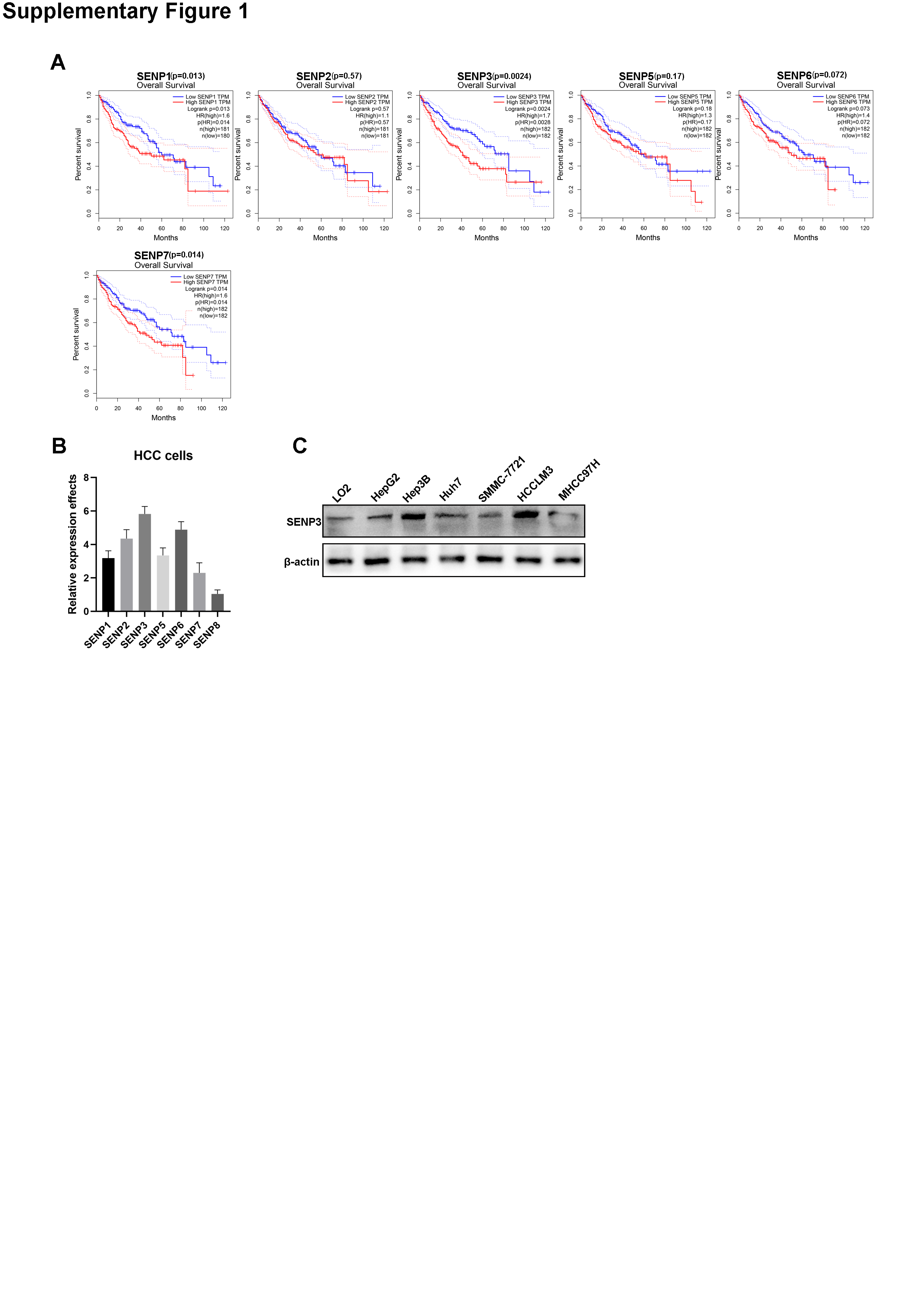

Supplement: Supplementary file 1 — Supplementary Figure 1 [file 41418_2024_1437_MOESM1_ESM.tif]

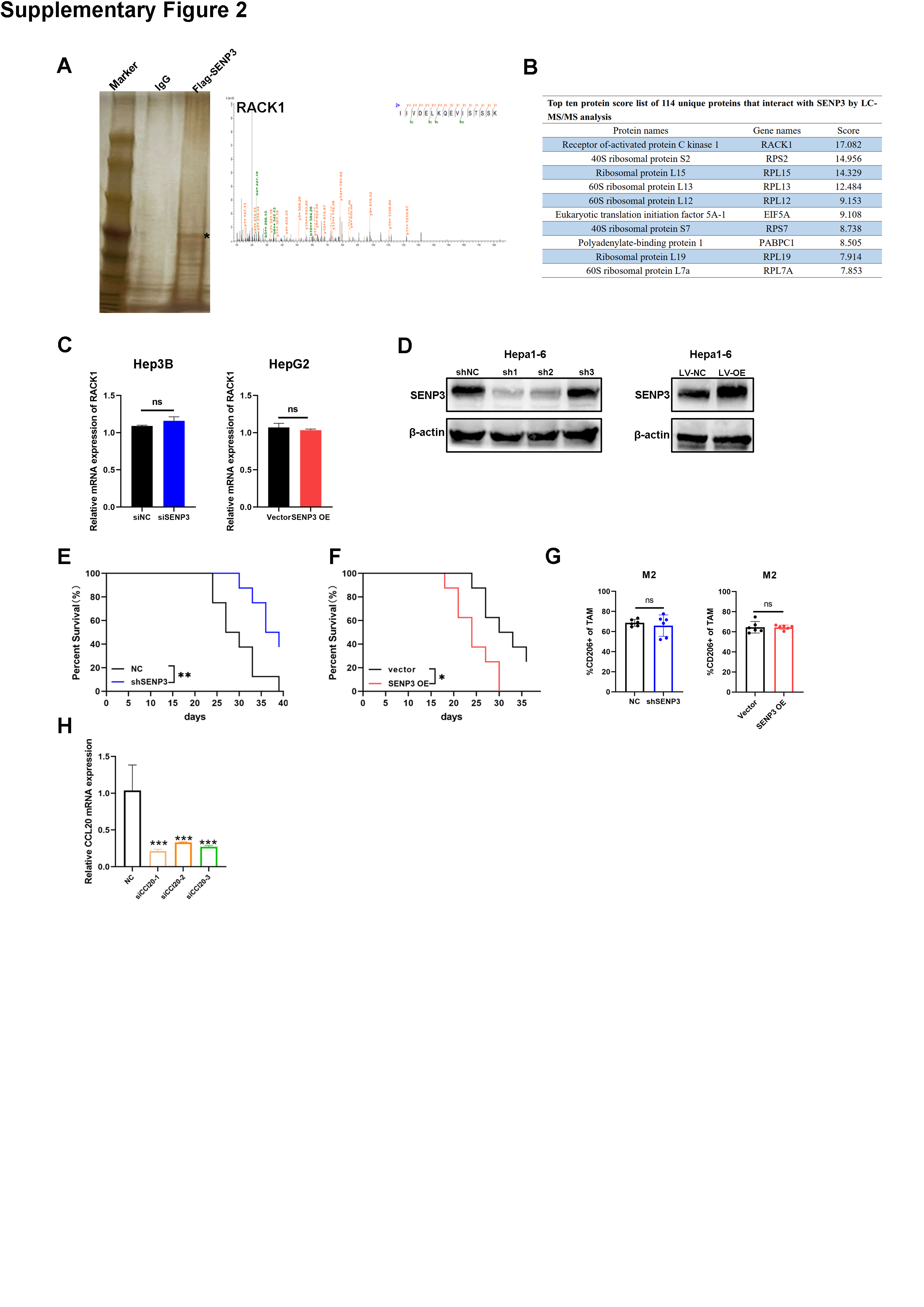

Supplement: Supplementary file 2 — Supplementary Figure 2 [file 41418_2024_1437_MOESM2_ESM.tif]

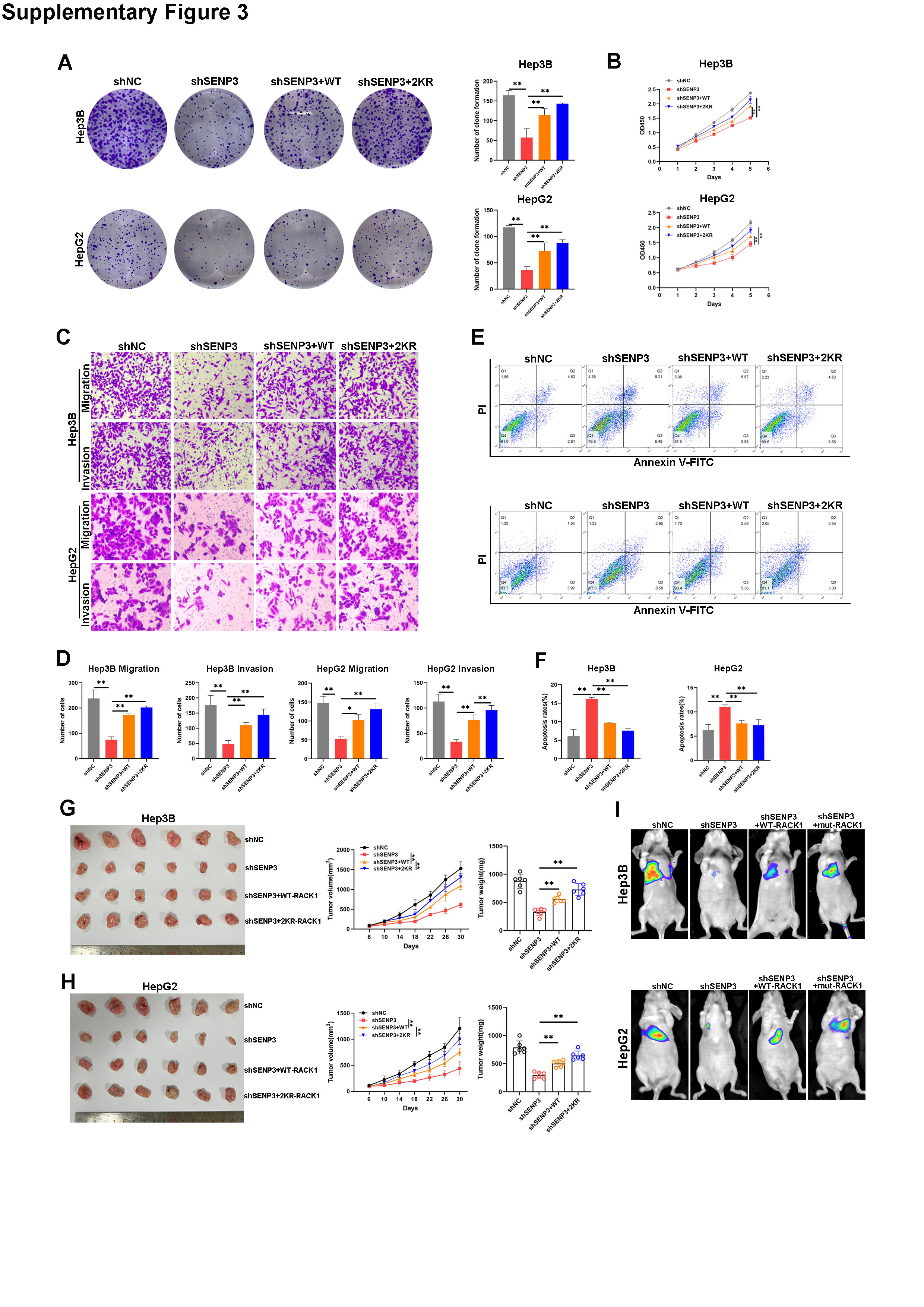

Supplement: Supplementary file 3 — Supplementary Figure 3 [file 41418_2024_1437_MOESM3_ESM.tif]

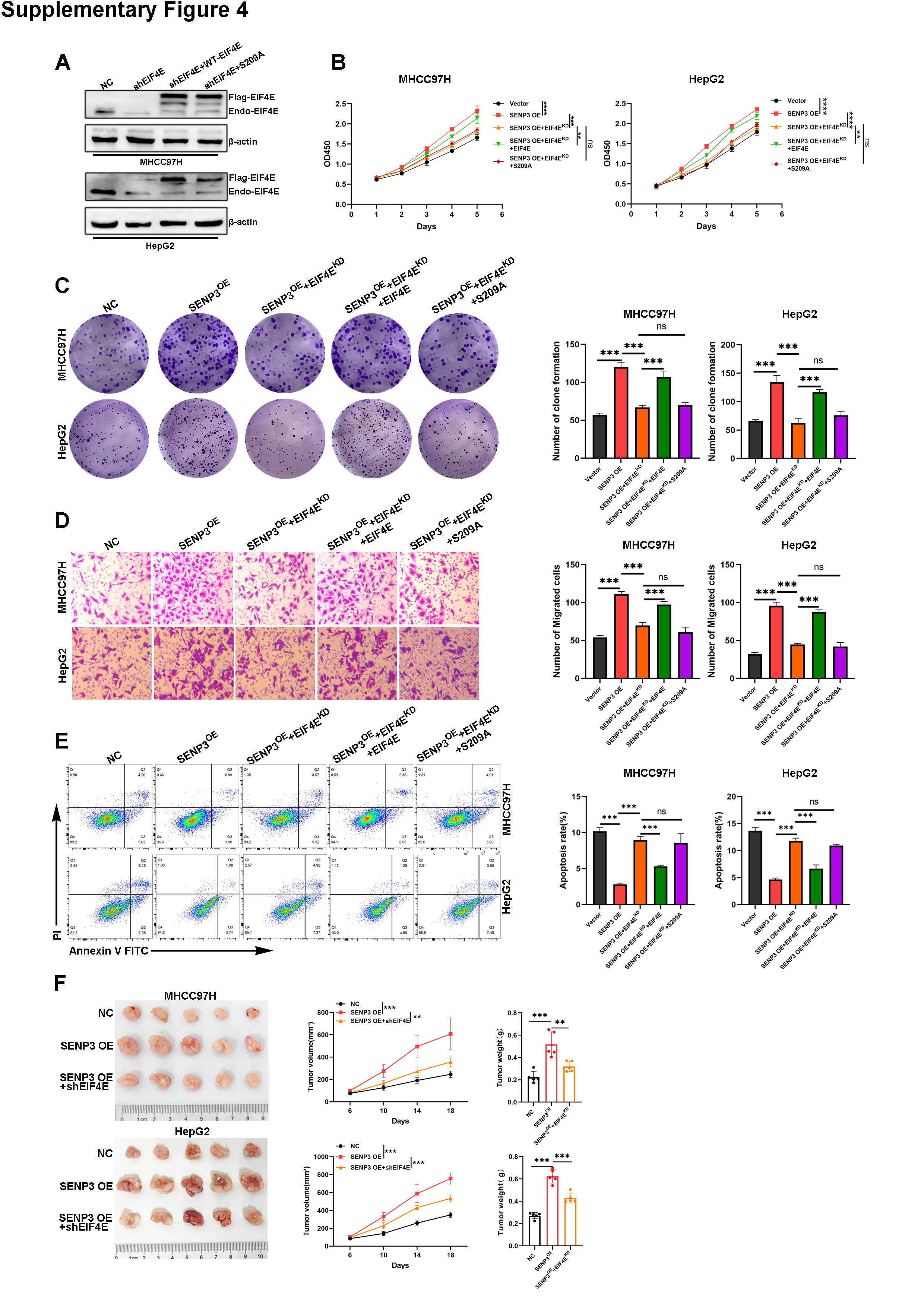

Supplement: Supplementary file 4 — Supplementary Figure 4 [file 41418_2024_1437_MOESM4_ESM.tif]

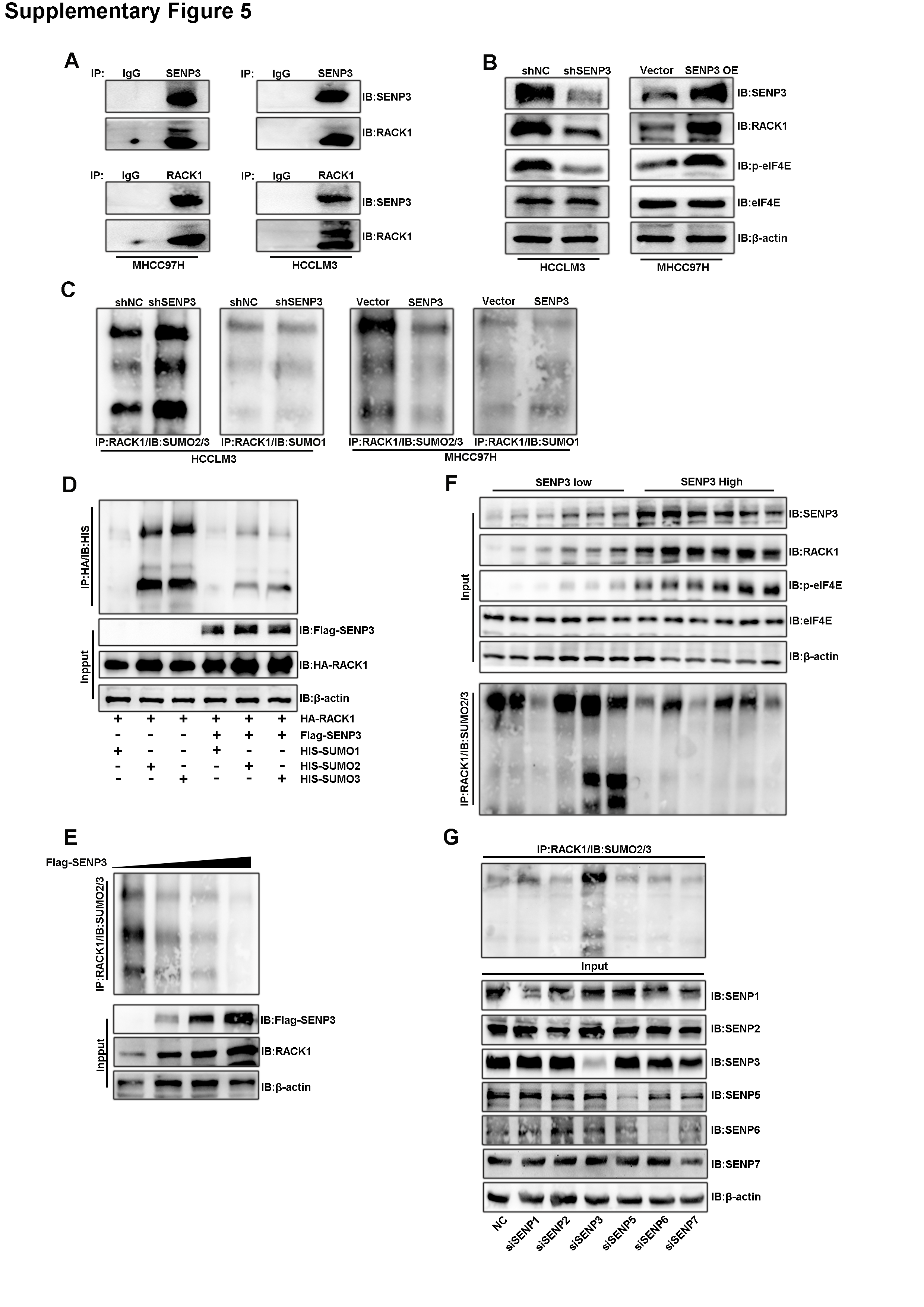

Supplement: Supplementary file 5 — Supplementary Figure 5 [file 41418_2024_1437_MOESM5_ESM.tif]

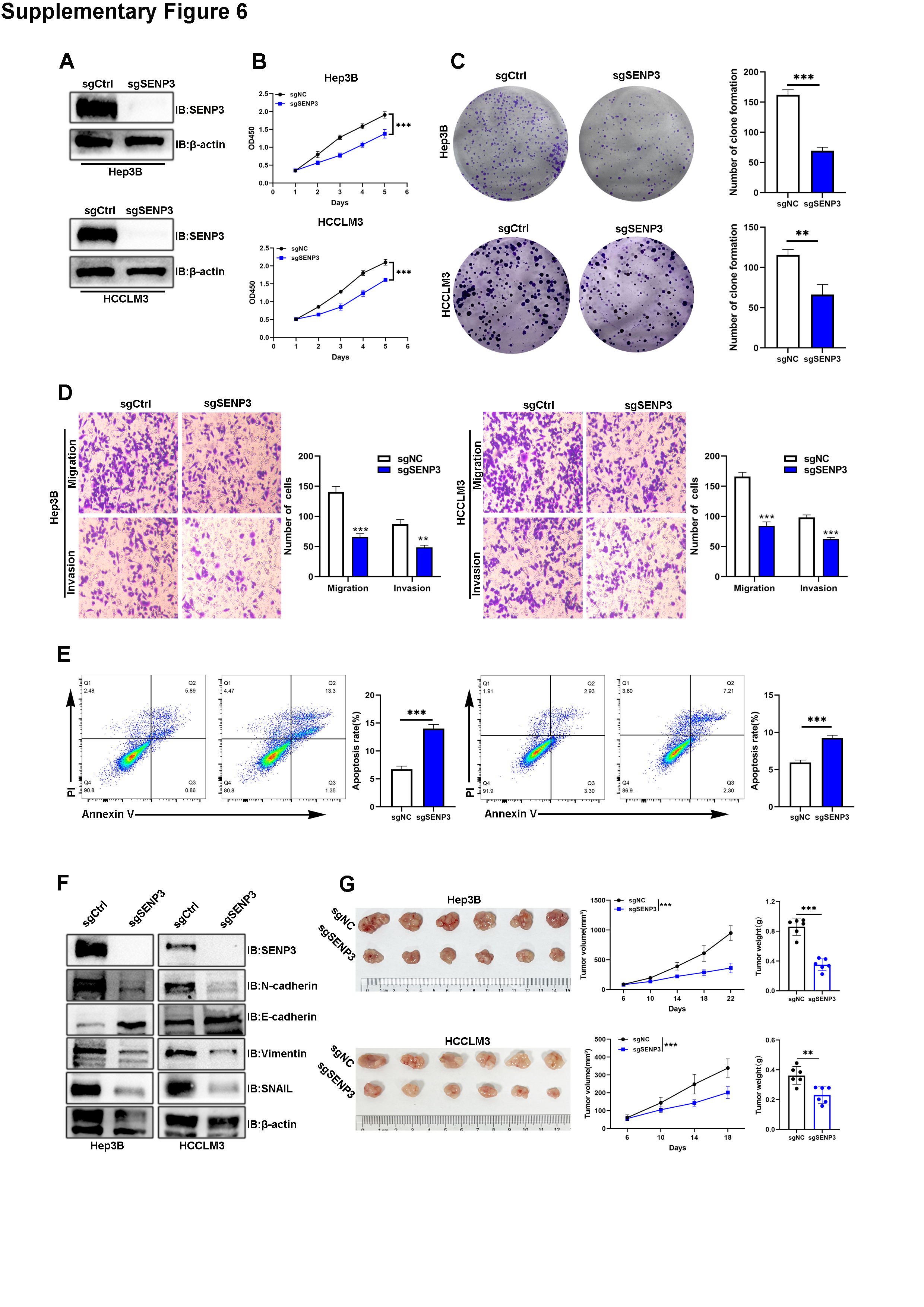

Supplement: Supplementary file 6 — Supplementary Figure 6 [file 41418_2024_1437_MOESM6_ESM.tif]

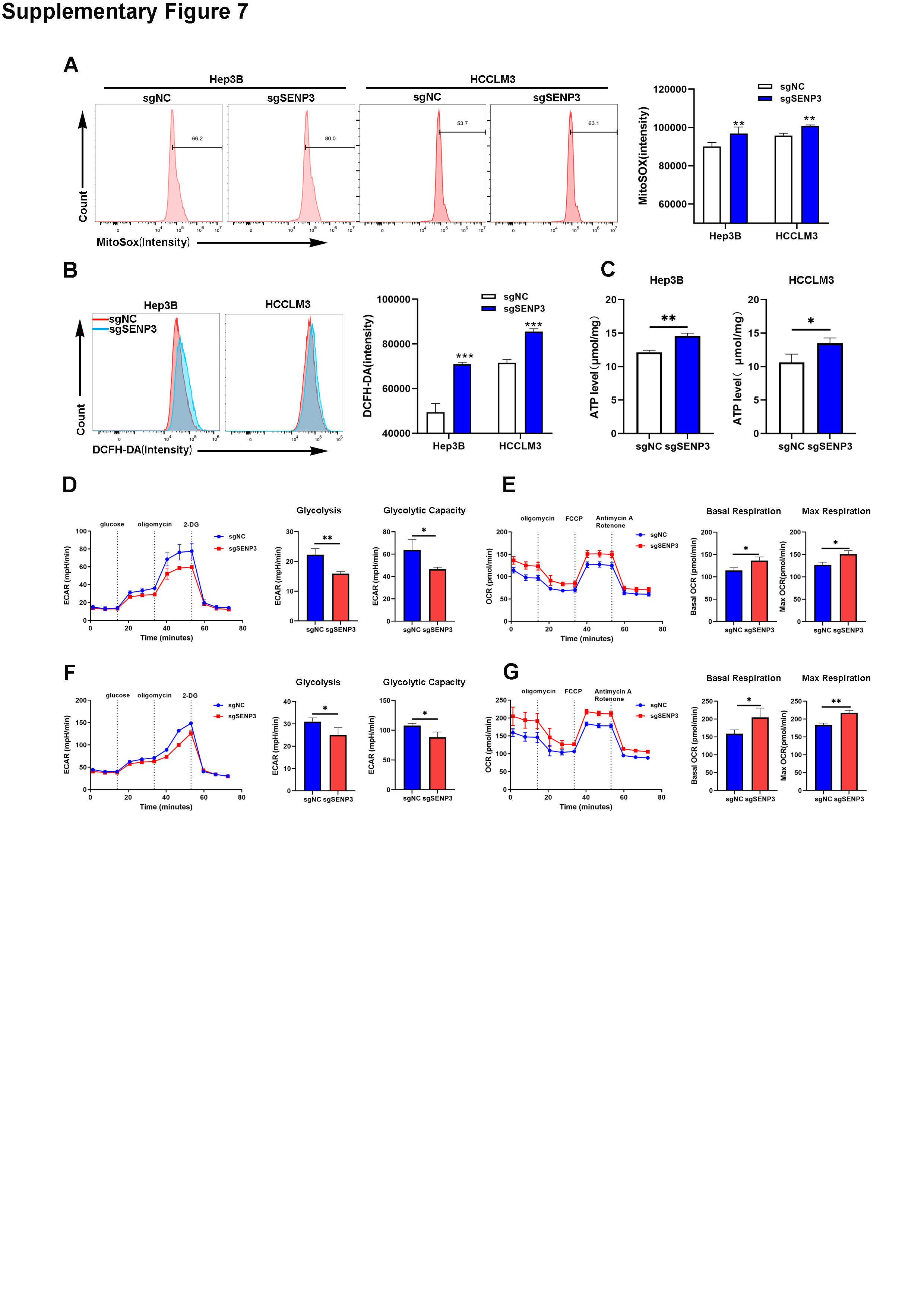

Supplement: Supplementary file 7 — Supplementary Figure 7 [file 41418_2024_1437_MOESM7_ESM.tif]

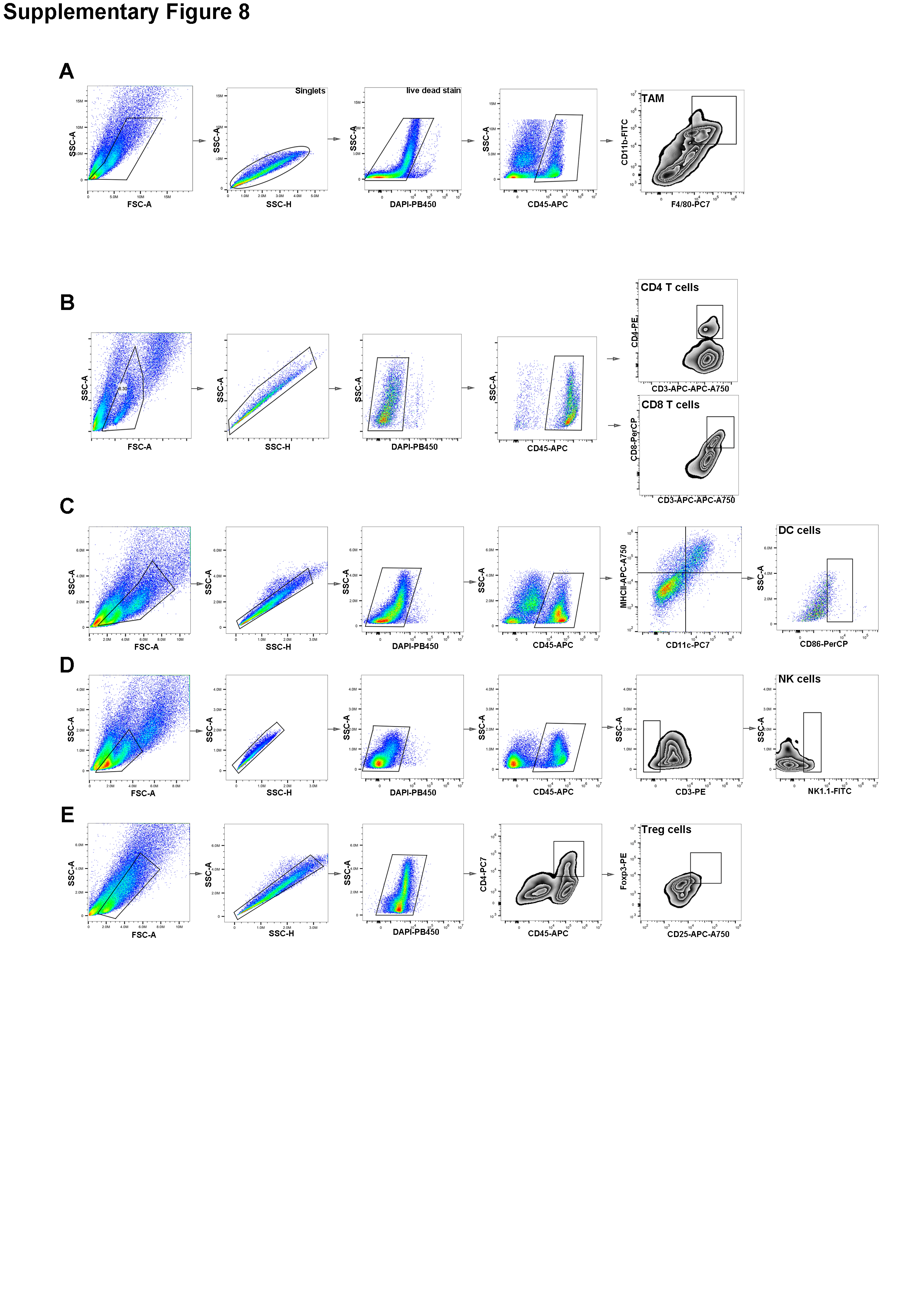

Supplement: Supplementary file 8 — Supplementary Figure 8 [file 41418_2024_1437_MOESM8_ESM.tif]
