# Supplementary material for: SENP3 inhibition suppresses hepatocellular carcinoma progression and improves the efficacy of anti-PD-1 immunotherapy
Source: Cell Death Differ. 2025 Jan 4;32(5):959–72. doi: 10.1038/s41418-024-01437-9 (PMC12089275; doi:10.1038/s41418-024-01437-9)
Supplement: Supplementary file 9 — Supplementary Information [file 41418_2024_1437_MOESM9_ESM.docx]

**Supplementary Materials and methods**

**HCC samples**

The human HCC samples were collected from patients underwent hepatectomy at the Affiliated Drum Tower Hospital, Medical School of Nanjing University. All the patients were informed the aim for collecting tissue samples and consented the collection. And this research had the approval of the ethics committee of Nanjing Drum Tower Hospital.

**Animal studies**

The 4-week-old C57BL/6 male mice and 4-week-old BALB/c nude mice were purchased from Gempharmatech for establishing the mouse models. The protocols of animal experiments had the approval of the Animal Care and Use Committee at Nanjing University.

To establish tumor xenograft model, appropriate number of HCC cells which stably expressing shSENP3 or SENP3 were injected subcutaneously into 4-week-old BALB/c nude mice.

To establish immune-competent mouse HCC model, 5×10^6^ SENP3 knockdown or overexpression Hepa1-6 cells were mixed with 100ul Matrigengel (ABW, Shanghai) firstly, the mixture was then injected subcutaneously into male C57BL/6 mice at 4 weeks of age.

To establish a lung metastasis model in null mice, 1×10^6^ luciferase-labeled Hep3B cells or MHCC97H cells were resuspended in 100ul PBS, and then PBS containing the cells was injected into 4-week-old BALB/c female nude mice through the tail vein.

For orthotopic xenograft tumor models, the subcutaneous tumor models were established firstly, then tumors were harvested and cut into 1 mm^3^ cubes. Next, five-week-old BALB/c nude mice were anesthetized with 2% isoflurane, then small pieces of tumors were implanted into left hepatic lobe. Three weeks after model establishment, mice were euthanized and livers were harvested for follow-up studies.

For macrophage depletion, mice were injected intraperitoneally with 50 mg/kg clodronate 5 days prior to tumor inoculation. Then mice were administrated with 25mg/kg clodronate every 5 days for 15 days. For ICB treatment, 200µg anti-PD-1 antibody or anti-IgG (Bio X cell) was injected intraperitoneally into hepa1-6-tumor bearing C57BL/6 mice every 4 days for three times.

To establish chemically induced liver cancer, fourteen-day-old male C57BL/6 mice were initially injected intraperitoneally with N-diethylnitrosamine (DEN, MCE, 25 mg/kg). After two weeks, mice received weekly intraperitoneal injections of 0.5 µL/g carbon tetrachloride (CCl4, Sigma, dissolved in olive oil). After 26 weeks of CCl4 administration, the DEN-CCl4 treated mice were sacrificed and the livers were harvested. The gross appearance of HCC was photographed, and number of grossly visible tumors and maximum tumor diameters were recorded in each liver of the DEN-CCl4-induced HCC mouse models.

**Cell lines and Cell Culture**

The human HCC cell lines (Hep3B, MHCC97L, Huh7, HepG2, MHCC97H, HCCLM3), HEK293T cells, and the mouse HCC cell line (hepa1-6 cells) were purchased from the Shanghai Institutes for Biological Sciences, Chinese Academy of Sciences (Shanghai, China). Cells were cultured in Dulbecco's modified Eagle's medium (DMEM, Gibco, USA) supplemented with 10% fetal bovine serum (FBS, Sigma, USA) at 37°C in a humidified incubator with 5% CO_2_. To obtain BMDMs, bone marrow cells were cultured in DMEM containing 10% FBS, 20ng/ml M-CSF (sinobiological, Beijing) for 7 days. All the culture mediums were added with 1% µg/ml penicillin-streptomycin (Gbico, USA).

**Small Interfering RNA, plasmid and lentivirus transfection**

The small interfering RNA was synthesized by GenePharma (Shanghai). The lentivirus encoding shSENP3, SENP3, wild-type RACK1, double mutant RACK1, sgSENP3-Cas9 and sheIF4E were synthesized by Genechem (Shanghai). The plasmids encoding wild-type RACK1, mutant RACK1 (K212R-RACK1, K264R-RACK1, K271R-RACK1, the corresponding Lys (K) residue was replaced with Arg (R)), wild-type eIF4E and S209A eIF4E were synthesized by Genechem (Shanghai). The small interfering RNA or related plasmid was transfected into cells using Lipofectamine 3000 according to the manufacturer’s introductions (Invitrogen, USA). The lentivirus was transfected into HCC cell lines, and puromycin (5ug/ml, Beyotime, China) was used to acquire stable cell lines expressing the related gene.

All the detailed shRNA/siRNA/sgRNA sequences were shown in Table S2.

The murine shSENP3-TBG-AAV8(AAV8-shSENP3)/negative control AAV8(1-1.5*10^12^ vg/ml) was synthesized by HanBio Technology (China), the virus was injected into mice via the tail vein at the 8th and 16th weeks during the chemically induced HCC models (5×10^11^vg/mouse).

**Proliferation assays.**

For the Cell Counting Kit-8 (CCK-8) assays, HCC cells transfected with siRNAs or plasmids were seeded into a 96-well plate at a density of 1000 cells per well. At each time point, the wells were treated with CCK-8 solution (Vazyme, Nanjing) and incubated in a cell incubator for 2 h. The microplate Spectrophotometer (Bio-Rad, California, USA) was used to measure the absorbance at OD450. For the clone formation assay, cells were seeded into a 6-well plate at a density of 500 cells per well and cultured for 14 days. Then the cells were washed with PBS and fixed with 4% paraformaldehyde. The fixed cells were stained with a solution of crystal violet (0.1%, Beyotime) for 30 minutes. For Edu assays, the CellLight EdU Apollo567 In Vitro Kit (RiboBio, China) was purchased and used according to the manufacturer's instructions.

**Transwell assays and Annexin V-FITC/PI Apoptosis Detection assays**

The Transwell inserts (Corning) with or without Matrigel were used to measure the ability of HCC cells to migrate and invade. Briefly, 5 × 10^4^ HCC cells are resuspended in DMEM without FBS, seeded into the upper chamber of the transwell inserts, and DMEM containing 10% FBS is added to the lower chamber. After 48 hours of culture, the cells in the upper chamber were removed and the migrated cells were stained with 0.1% crystal violet.

For Apoptosis detection assays, the cells and culture media were harvested. After centrifugation, the cells were stained with AnnexinV-FITC and PI (Vazyme, Nanjing) for 15 minutes. The apoptotic cells were detected using a flow cytometer (FACSCalibur, BD).

**Co-immunoprecipitation (co-IP) assay and Mass spectrometry Analysis**

Total proteins were first extracted from the cells transfected with related plasmids or lentiviruses. Then the proteins were incubated with normal IgG antibodies (rabbit or mouse, BD, USA) and 20ul Protein A/G PLUS-Agarose (Santa Cruz, USA) for 2h. After removal of non-specific binding, the proteins were incubated with relevant primary antibodies overnight at 4℃. Finally, the Agarose was used to acquire immuno-precipitates for further studies.

The HepG2 cells were transfected with plasmids encoding Flag-SENP3, then the Flag antibodies were used in Co-IP assays for acquiring Flag-SENP3 proteins, next the protein mixtures were subject to SDS-PAGE (Beyotime). The existence of proteins was confirmed by silver staining. Last, the Applied protein technology corporation (China, Shanghai) performed the analysis of Mass spectrometry on the gels that we send.

**Cytokines array assays and ELISA**

To detect the changes of cytokines secreted by hepa1-6 cells, the Proteome Profiler Mouse XL Cytokine Array (ARY028) was purchased from R&D corporation. The prepared membrane arrays were first incubated with the test samples at 4℃ overnight. Next, the membrane arrays were washed and incubated with Detection Antibody Cocktail and Streptavidin-HRP respectively. Finally, the membrane arrays were incubated with Chemi Reagents 1 and 2 for 2 minutes and detected using a Tanon 5200 Chemiluminescent Imaging System (Tanon, China).

The Hepa1-6 cells, with or without SENP3 knockdown, were cultured in serum-free DMEM for 24 hours. Afterward, the culture supernatants were collected and centrifuged for 20 minutes. The amount of secreted CCL20 was measured using the mouse MIP3 alpha (CCL20) ELISA Kit (ab100728, Abcam).

**Western Blotting and Quantitative real-time PCR (qRT-PCR)**

The RIPA Lysis Buffer (Beyotime) containing 1% protease inhibitors (ncmbio, China) and 1% PMSF (Beyotime) was used to extract the proteins from cells or tumor tissues.

The protein concentration was measured with the BCA kit (Vazyme, Nanjing) prior to protein denaturation. The denatured proteins underwent SDS-PAGE and were then transferred from the gels onto polyvinylidene difluoride (PVDF) membranes (Millipore,USA). After blocking with 5% skim milk for 2 hours, the membranes were incubated with primary antibodies at 4℃ overnight. Subsequently, the membranes were washed with TBST three times and incubated with secondary antibodies for 2 hours. The signals of membranes were detected using an ECL kit (ncmbio, China) with a Tanon 5200 Chemiluminescent Imaging System (Tanon, China).

The TRIzol® reagent (Takara, USA) was used to extract total RNA as previously described. The cDNA was synthesized from RNA using HiScript II Q RT SuperMix (Vazyme). qRT-PCR was performed using the SYBR Green PCR kit (Vazyme). The mRNA expression levels were normalized to GAPDH and calculated using the 2^−ΔΔCq^ method. The primer sequences designed for the study are shown in Table S1.

**Measurement of intracellular and mitochondrial levels of reactive oxygen species (ROS) and Measurement of cellular ATP level**

The total ROS level in cells was determined by a flow cytometer using fluorescent dye 2′,7′-dichlorofluorescin diacetate (DCFH-DA, Beyotime, S0035). Briefly, cells with or without SENP3 were collected and washed with PBS, then cells were incubated with 5μM DCFH-DA at 37 °C for 30 min. After washing with DMEM three times, the Fluorescence was detected by FACS using a flow cytometer (Beckman Coulter Cytoflex S, Canada). To evaluate the mitochondrial level of ROS, the cells were collected and incubated with 200nM Mito-Tracker Red CMXRos (Beyotime, C1035) at 37°C for 30min. The fluorescent intensity was detected using a flow cytometer. ATP was measured using an ATP Assay Kit (Beyotime, S0026) according to the manufacturer’s instructions. Briefly, the cells were lysed with lysis buffer. Then, supernatants were obtained after centrifugation. Next, the substrate solutions were added into an opaque 96-well white plate. ATP standards and supernatant samples were added separately to the assay wells and allowed to react for 5 minutes at room temperature. A SpectraMaxID5 (Molecular Devices ID5) was used to detect the luminescence.

**Oxygen consumption rate (OCR) and extracellular acidification rate (ECAR)**

The HCC cells with or without SENP3 (1×10^4^) were seeded into a XF96 plate and cultured in a CO_2_ incubator overnight. A probe plate was hydrated in a non-CO2 incubator overnight.

To evaluate the glycolytic capacity, the cells were washed with test solutions (Seahorse XF Basal Medium containing with 2mM glutamine) firstly, then 25 μL each of 10 mM glucose, 1 μM oligomycin, and 100 mM 2-deoxyglucose (2-DG) were added to the probe, according to the manufacturer’s instructions.

For Cell Mito Stress Test, the cells were washed with test solutions (Seahorse XF Basal Medium containing 1 mM sodium pyruvate, 2 mM glutamine and 10 mM glucose), then 25 μL each of 1 μM oligomycin, 0.5 μM FCCP, and 1 μM rotenone were added into the probe, according to the manufacturer’s instructions. All reagents used for ECAR and OCR detection were purchased from Seahorse.

ECAR and OCR were determined by a XF96 Extracellular Flux Analyzer (Seahorse Bioscience) and normalized by the amount of total protein.

**Immunohistochemistry (IHC) and immunofluorescence (IF) staining**

For IHC staining, tumor tissues were first fixed with 4 % formalin overnight, after dehydrated with graded alcohols, the samples were embedded in paraffin blocks. The paraffin blocks were cut into 5um thin section and placed on a slide. After endogenous peroxidase inactivation and antigen retrieval, the tumor sections were blocked with animal nonimmune serum (Servicebio, China) and then incubated with primary antibodies at 4 °C overnight. After incubation with secondary antibodies, the cell nuclei were stained with hematoxylin (Servicebio). The Olympus BX51 microscope was used to photograph and count the positive areas.

For immunofluorescence staining, the 5×10^4^ HCC cells were seeded in a Confocal dish and cultured for 24h. The cells were fixed with 4% formalin and their permeability was enhanced with 0.5% Triton-X-100. After blocking with 1% BSA, the samples were incubated with primary antibodies available for IF overnight. After PBST washing, the samples were incubated with fluorescence-labeled secondary antibodies (SA00013-1, SA00013-4, proteintech) for primary antibody binding. The fluorescence signals were detected with a laser scanning confocal microscope (Olympus FV3000, Japan).

**Multicolor Flow cytometry**

To analyze immune cell infiltration in tumor tissues, tumor samples were cut into pieces and then digested with collagenases (Sigma) for 2 hours on a horizontal shaker at 37℃. Next, the cells were passed through 70 µm nylon filters (Nest) for removing undigested pieces. After centrifugation, the precipitated cells were treated with Red Blood Cell Lysis Buffer (Beyotime) to remove red blood cells. After that, the cells were adjusted to a concentration of 2×10^7^ cells/mL and then blocked with 2% BSA for 30 minutes. Next, 100µl cell suspensions were moved to the flow tube and the surface markers were stained with corresponding fluorescent antibodies for 15 min at room temperature. For intracellular staining, eBioscience™ Flow Intracellular Fixation & Permeabilization Buffer Set were used to enhance the cell permeability, then the cells were stained with fluorescent antibodies for 30min. Then the fluorescent signals were measured by a flow cytometer. The data obtained from flow cytometry was analyzed using FlowJo software (FlowJo v10.1.8.1).

The antibodies used in multicolor flow Cytometry included APC anti-mouse CD45 (#147707), FITC anti-mouse CD11b (#101205), PE/Cyanine7 anti-mouse F4/80 (#123113), APC/Fire™ 750 anti-mouse CD3(#100247), PerCP anti-mouse CD8a (#100731), PE anti-mouse CD4 (#100407), PE/Cyanine7(PC7) anti-mouse CD11c, APC/Fire™ 750 anti-mouse I-A/I-E(#107651) PerCP anti-mouse CD86(#105025), FITC anti-mouse NK-1.1(#156507), PE/Cyanine7 anti-mouse CD4(#100421), PE anti-mouse FOXP3 (#126403), APC/Fire™ 750 anti-mouse CD25 antibodies. The antibodies listed above were provided by Biolegend Corporation.

**Polysome profiling**

To measure the translation efficiency of CCL20, polysome profiling was performed as described previously (PMID: 29224064). Hepa1-6-shNC and Hepa1-6-shSENP3 cells were initially cultured for 24 hours in DMEM supplemented with 10% FBS. Next, the 10^7^ cells were treated with cycloheximide (100 μg/mL) for 15 minutes. The cells were then digested with 0.25% trypsin-EDTA solution, collected, and washed three times with cold PBS containing 100 μg/mL of cycloheximide. Cell pellets were lysed in 500 µL Polysome Profiling Lysis Buffer (epibiotek, China) for 30 minutes on ice.  The supernatant was collected after centrifugation and loaded onto a 10%–50% sucrose density gradient. Polysome sedimentations were acquired after centrifugation at 36,000 rpm using SW41 rotor for 3 h at 4°C. After that, the automated fractionation machine (Piston Gradient Fractionator™ Biocomp Instruments) was used to fractionate the sucrose of different densities into 18 tubes for further study.

**Chemotaxis assays**

The transwell inserts (8µm, Milipore) were used to perform chemotaxis assays. Hepa1-6 cells were cultured for 24 hours, then the culture medium was collected and added to the lower chamber, the upper chambers of the inserts were seeded with 5×10^4^ BMDMs (70% purity). After culturing for 24h, the macrophages in the bottom compartment were stained with 0.1% crystal violet solution for 15min, and the migrated macrophages were photographed and counted under a microscope.

**Statistical analysis**

The GraphPad Prism 8.0 was used to perform all the statistical tests, and p value <0.05 was considered statistical significance (*p < 0.05; **p < 0.01; ***p <0.001, ****p <0.0001).

The unpaired two-tailed Student's t-test was used to analyze differences between two groups. One-way or two-way analysis of variance (ANOVA) was used to analyze differences between multiple groups, and the log-rank test was used to analyze differences in survival curves. The data were presented as mean ± SD, and n values are expressed in the figure legends.

**Supplementary Table S1. Primers for qPCR**

| **qPCR Primers** | | |
| --- | --- | --- |
| Gene | | Sequence (5’to 3’) |
| hSENP3 | F | GGATGCTGCTCTACTCAAAAAGC |
|  | R | GGGAGTCAAAACGACAACAGG |
| hGAPDH | F | GGAGCGAGATCCCTCCAAAAT |
|  | R | GGCTGTTGTCATACTTCTCATGG |
| hRACK1 | F | TGGGATGGAACCCTGCG |
|  | R | GTATGGCCCACCAATCGCC |
| hCCL20 | F | TGCTGTACCAAGAGTTTGCTC |
|  | R | GCACACAGACAACTTTTTCTTT |
| mCCL20 | F | GCCTCTCGTACATACAGACGC |
|  | R | CCAGTTCTGCTTTGGATCAGC |
| mGAPDH | F | AGGTCGGTGTGAACGGATTTG |
|  | R | TGTAGACCATGTAGTTGAGGTCA |

**Supplementary Table S2. The shRNA/siRNA/ sgRNA sequences in the study.**

| For shRNA construction |  |
| --- | --- |
| human-shSENP3-1 | 5'-CACCAGGGCUGGAAAGGUU-3' |
| human-shSENP3-2 | 5'-CUGGCCCUGUCUCAGCCAU-3' |
| mus-shSENP3-1 | 5’-GCAGAGTATCTTAGATGAATT-3’ |
| mus-shSENP3-2 | 5’-GCCAGCATACTCATCAGTAAT-3’ |
| mus-shSENP3-3 | 5’-CCTAAGCATATTGCCAAGTAT-3’ |
| mus-siCCl20-1 | 5’-GACUGUUGCCUCUCGUACATT-3’ |
| mus-siCCl20-2 | 5’-GCUUGUGACAUUAAUGCUATT-3’ |
| mus-siCCl20-3 | 5’-GCCUAAGAGUCAAGAAGAUTT-3’ |
| human-sgRNA-3 | 5’-AGATTGTCCCCCAAAACCGT-3’ |
| human-siSENP1 | 5’- CAAGAAGUGCAGCUUAUAATT-3’ |
| human-siSENP2 | 5’-GGGUAAUAAAUCUCCUAAUTT-3’ |
| human-siSENP5 | 5’- GGGAGTGTACAGAGCTGATTCATGA-3’ |
| human-siSENP6 | 5’- GGACAA AUCUGCUCAGUGU-3’ |
| human-siSENP7 | 5’-GGCUCACAACGAAGUAAGATT-3’ |
| sh-eIF4E | 5’-CCGGCCACTCTGTAATAGTTCAGTACTCGAGTACTGAACTATTACAGAGTGGTTTTTG-3’ |

**Supplementary Table S3. Antibodies and Reagents used in this study.**

| Antibody/Reagent | Specie | Source | Identify |
| --- | --- | --- | --- |
| SENP3 | Rabbit | CST | Cat#5591 |
| RACK1 | Mouse | Santa Cruz | Cat#sc17754 |
| eIF4E | Rabbit | abcam | Cat #ab33766 |
| p-eIF4E | Rabbit | abcam | Cat #ab76256 |
| Flag | Rabbit | CST | Cat #14793 |
| Flag | Mouse | CST | Cat #8146 |
| HIS  HA  HA  SUMO2/3  SUMO1  BCL2  Snail  Cyclin D1  ubiquitin  Ki67  CD68  CD8  β-actin  SENP1  SENP2  SENP5  SENP6  SENP7  N-Cadherin  E- Cadherin  Vimentin  CoraLite488-conjugated Goat Anti-Mouse IgG  CoraLite594 – conjugated Goat  Anti-Rabbit IgG  Mouse Anti-Rabbit IgG (Light-Chain Specific)  Rabbit Anti-Mouse IgG (Light Chain Specific)  HRP-conjugated Goat Anti-Rabbit IgG  HRP-conjugated Goat Anti-Mouse IgG  Cycloheximide  MG132  Clodronate Liposomes | Rabbit  Rabbit  Mouse  Rabbit  Rabbit  Rabbit  Rabbit  Rabbit  Rabbit  Rabbit  Rabbit  Rabbit  Mouse  Rabbit  Rabbit  Rabbit  Rabbit  Mouse  Rabbit  Rabbit  Rabbit  Goat  Goat  Goat  Goat  Goat  Goat | CST  Proteintech  Proteintech  CST  CST  CST  CST  CST  Proteintech  Servicebio  Servicebio  Servicebio  Proteintech  CST  Proteintech  Proteintech  Affinity  Santa Cruz  CST  CST  CST  Proteintech  Proteintech  CST  CST  Proteintech  Proteintech  MCE  Beyotime  Yesen | Cat #12698  Cat #51064-2-AP  Cat #66006-2-Ig  Cat #4971  Cat #4930  Cat #3498  Cat #3879  Cat #2922  Cat #10201-2-AP  Cat #GB121141  Cat #GB113109  Cat #GB114196  Cat #66009-1-Ig  Cat #11929  Cat #29772-1-AP  Cat #19529-1-AP  Cat #AF0277  Cat #sc-373821  Cat #13116  Cat #3195  Cat #5741  Cat #SA00013-1  Cat # SA00013-4  Cat #93702  Cat #58802  Cat #SA00001-1  Cat #SA00001-2  Cat #HY-12320  Cat #S1748  Cat #40337ES |

| **Supplementary Table S4.** Correlations between SENP3 expression and clinical characteristics in HCC patients (n = 100) | | | | |
| --- | --- | --- | --- | --- |
| Characteristics | Number | SENP3 expression | | P-value |
|  |  | Low group | High group |  |
| Age(years) |  |  |  |  |
| <50 | 25 | 15 | 10 | 0.248 |
| ≥50 | 75 | 35 | 40 |  |
| Gender |  |  |  |  |
| FeMale | 29 | 13 | 16 | 0.509 |
| Male | 71 | 37 | 34 |  |
| Cirrhosis  Present  Absent  HBV infection  Positive  Negative  Tumor size(cm) | 87  13  82  18 | 43  7  39  11 | 44  6  43  7 | 0.766  0.298 |
| <5 | 44 | 31 | 13 | **<0.001**^***^ |
| ≥5 | 56 | 19 | 37 |  |
| Microvascular invasion  Present  Absent | 46  54 | 17  33 | 29  21 | **<0.05**^*^ |
| Tumor multiplicity  Simple  Multiple | 73  27 | 40  10 | 33  17 | 0.115 |
| α-fetoprotein (ng/ml)  ≤20  >20  TNM stage | 43  57 | 20  30 | 23  27 | 0.545 |
| I | 57 | 34 | 23 | **<0.05**^*^ |
| II/III | 43 | 16 | 27 |  |
| Edmonson stage |  |  |  |  |
| I/II | 81 | 46 | 35 | **<0.01**^**^ |
| III/IV | 19 | 4 | 15 |  |

| *P<0.05, **P<0.01, ***P<0.001 |
| --- |

**Supplementary Figure legends**

**Supplementary Figure 1. SENP3 is highly expressed in HCC cell lines and is associated with a poor prognosis in HCC patients.**

(A) Analysis of the Kaplan‒Meier overall survival curves of HCC patients with different SENP expression levels (based on the GEPIA database). (B) DEPMAP analysis of the levels of SENP family members in 24 HCC cell lines. (C) SENP3 protein expression in common HCC cell lines was evaluated by Western blotting (n=3).

**Supplementary Figure 2. SENP3 is closely associated with RACK1 in HepG2 cells.**

(A) Flag-SENP3-overexpressing HepG2 cells were subjected to co-IP by using control IgG or an anti-Flag antibody conjugated with magnetic beads. Then, the precipitates were subjected to silver staining (left panel). The unique peptide spectrum of RACK1 is shown in the right panel. (B) The top ten proteins associated with Flag-SENP3 in HepG2 cells. (C) The mRNA levels of RACK1 in HCC cells after SENP3 knockdown or overexpression. (D) The efficiency of SENP3 knockdown or overexpression in Hepa1-6 cells was confirmed by Western blotting (n=3). (E) OS of Hepa1-6 tumor-bearing C57BL/6 mice in the NC or shSENP3 group (n=8). (F) OS of Hepa1-6 tumor-bearing C57BL/6 mice in the vector or SENP3 OE group (n=8). (G) Statistical analysis of M2 ratio in TAMs from different groups according to the flow cytometry results (n=6). (H) The efficiency of CCL20 knockdown was evaluated by RT‒PCR analysis. Two-tailed unpaired Student’s t test and log-rank test were performed to determine significance. *P < 0.05, **P < 0.01, ***P < 0.001, ****P < 0.0001.

**Supplementary Figure 3**. **SENP3 promotes HCC malignancy in a RACK1-dependent manner**

SENP3-knockdown HCC cells were transduced with WT-RACK1 or the RACK1 double mutant lentivirus for further studies. (A-B) The proliferation abilities of the four treatment groups were evaluated by CCK-8 and colony formation assays (n=3). (C-D) The migration and invasion abilities of the cells in the four treatment groups were evaluated by Transwell assays (n=3). (E-F) The apoptosis of HCC cells in the four different groups was evaluated by flow cytometry using Annexin V-FITC/PI staining (n=3). (G-H) Representative images of xenograft tumors in nude mice from the four treatment groups. Tumor weights and tumor volumes were recorded in this study (n=6). (I) Representative bioluminescence images of nude mice from the four treatment groups (n=5). One-way ANOVA was used to determine significance. *P < 0.05, **P < 0.01, ***P < 0.001.

**Supplementary Figure 4**. **SENP3 promotes HCC malignancy in an eIF4E-dependent manner**

The eIF4E expression was silenced firstly in SENP3-overexpression HCC cells, and then the cells were transduced with wild-type eIF4E or S209A eIF4E plasmids. (A) The efficiency of eIF4E knockdown and exogenous eIF4E overexpression were confirmed by Western blot (n=3). (B-C) The proliferation abilities of the five treatment groups were evaluated by CCK-8 and colony formation assays (n=3). (D) The migration abilities of the cells in the five treatment groups were evaluated by Transwell assays (n=3). (E) The apoptosis of HCC cells in the five different groups was evaluated by flow cytometry using Annexin V-FITC/PI staining (n=3). (F) Representative images of nude mouse HCC xenograft tumors in different groups. The tumor volumes and tumor weights are shown in the right panel (n=5). One-way ANOVA was used to determine significance. *P < 0.05, **P < 0.01, ***P < 0.001.

**Supplementary Figure 5. RACK1 was primarily modified by SUMO2/3 and specifically deSUMOylated by SENP3.** (A) Co-IP and Western blot analyses were performed to confirm the interaction between SENP3 and RACK1 in HCCLM3 and MHCC97H cells. (B) The protein levels of RACK1, p-eIF4E, and eIF4E were evaluated by Western blotting in HCCLM3 and MHCC97H after knockdown or overexpression of SENP3. (C) Co-IP and Western blot assays were performed to determine SUMO1 and SUMO2/3 modification of RACK1 in HCCLM3 and MHCC97H cells after SENP3 knockdown or overexpression. (D) HEK293T cells were transfected with HA-RACK1, Flag-SENP3 and HIS-SUMO1 or HIS-SUMO2 or HIS-SUMO3. HA-RACK1 SUMOylation assays were performed using HA antibodies. (E) HepG2 cells were transfected with HA-RACK1 and increasing amounts of Flag-SENP3 for 48h. RACK1 SUMOylation was determined by co-IP and Western blot assays. (F) The frozen HCC tissues were divided into SENP3 high group or SENP3 low group based on SENP3 protein level. The proteins were extracted from tissues and co-IP assays were performed by using RACK1 antibodies. The SUMOylation of RACK1, SENP3, p-eIF4E and eIF4E were examined by Western blot assays. (G) SENP1, SENP2, SENP3, SENP5, SENP6 or SENP7 were knocked down in HepG2 cells using the corresponding siRNAs. Cell lysates were prepared for precipitation with RACK1 antibodies, and SUMOylation was detected by SUMO2/3 antibodies. All assays were performed in triplicate, and the results of representative experiments are shown.

**Supplementary Figure 6. SENP3 deletion inhibits HCC cell proliferation, invasion and metastasis in vitro and in vivo.**

(A) The efficiency of SENP3 knockout in Hep3B and HCCLM3 cells was evaluated by Western blotting (n=3). (B-C) The proliferation of Hep3B and HCCLM3 after SENP3 deletion was assessed by CCK8 assay and colony formation assay (n=3). (D) The migratory and invasive capacity of HCC cells with SENP3 deletion was evaluated by using a Transwell assay (n=3). (E) The apoptosis of HCC cells with SENP3 deletion was evaluated by flow cytometry using the Annexin V FITC/PI staining method (n=3). (F) The protein level of snail, Vimentin, and N-cadherin and E-cadherin were evaluated by Western blotting in HCCLM3 and Hep3B after knockout of SENP3 (n=3). (G) Representative images of nude mouse HCC xenograft tumors in different groups. The tumor volumes and tumor weights are shown in the right panel (n=6). Two-tailed unpaired Student's t tests were used to determine significance. *P < 0.05, **P < 0.01. ***P < 0.001.

**Supplementary Figure 7. SENP3 deletion decreases HCC cell glycolysis.**

(A-B) The intracellular and mitochondrial levels of reactive oxygen species (ROS) in HCC cells were measured by flow cytometry using DCFH-DA and MitoTrackerTM Red CMXRos, respectively (n=3). (C) The cellular ATP levels in HCC cells after SENP3 knockout were detected by using an ATP Assay Kit (n=3). (D-G) Extracellular acidification rate (ECAR) and Oxygen consumption rate (OCR) were determined by Seahorse analysis in control and SENP3 deletion HCC cells (n=3). Two-tailed unpaired Student's t tests were used to determine significance. *P < 0.05, **P < 0.01. ***P < 0.001.

**Supplementary Figure 8. Gating strategies used for flow cytometry analysis.**

Gating strategy used to sort TAMs (A), CD8+ T cells (B), CD4+ T cells (B), DCs (C), NK cells (D) and Tregs (E).
