# Supplementary material for: SENP3 inhibition suppresses hepatocellular carcinoma progression and improves the efficacy of anti-PD-1 immunotherapy
Source: Cell Death Differ. 2025 Jan 4;32(5):959–72. doi: 10.1038/s41418-024-01437-9 (PMC12089275; doi:10.1038/s41418-024-01437-9)

Uncropped original western blots

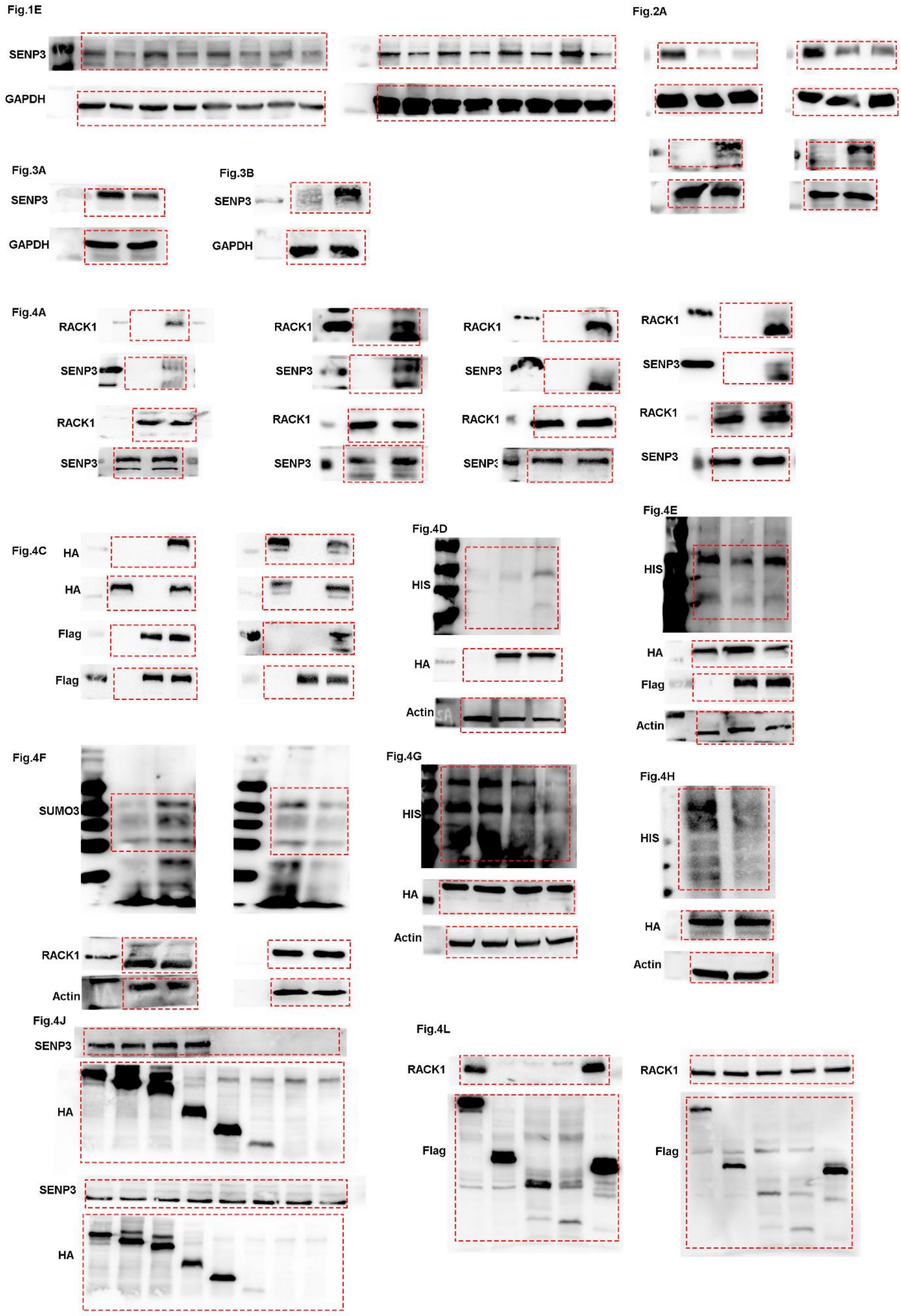

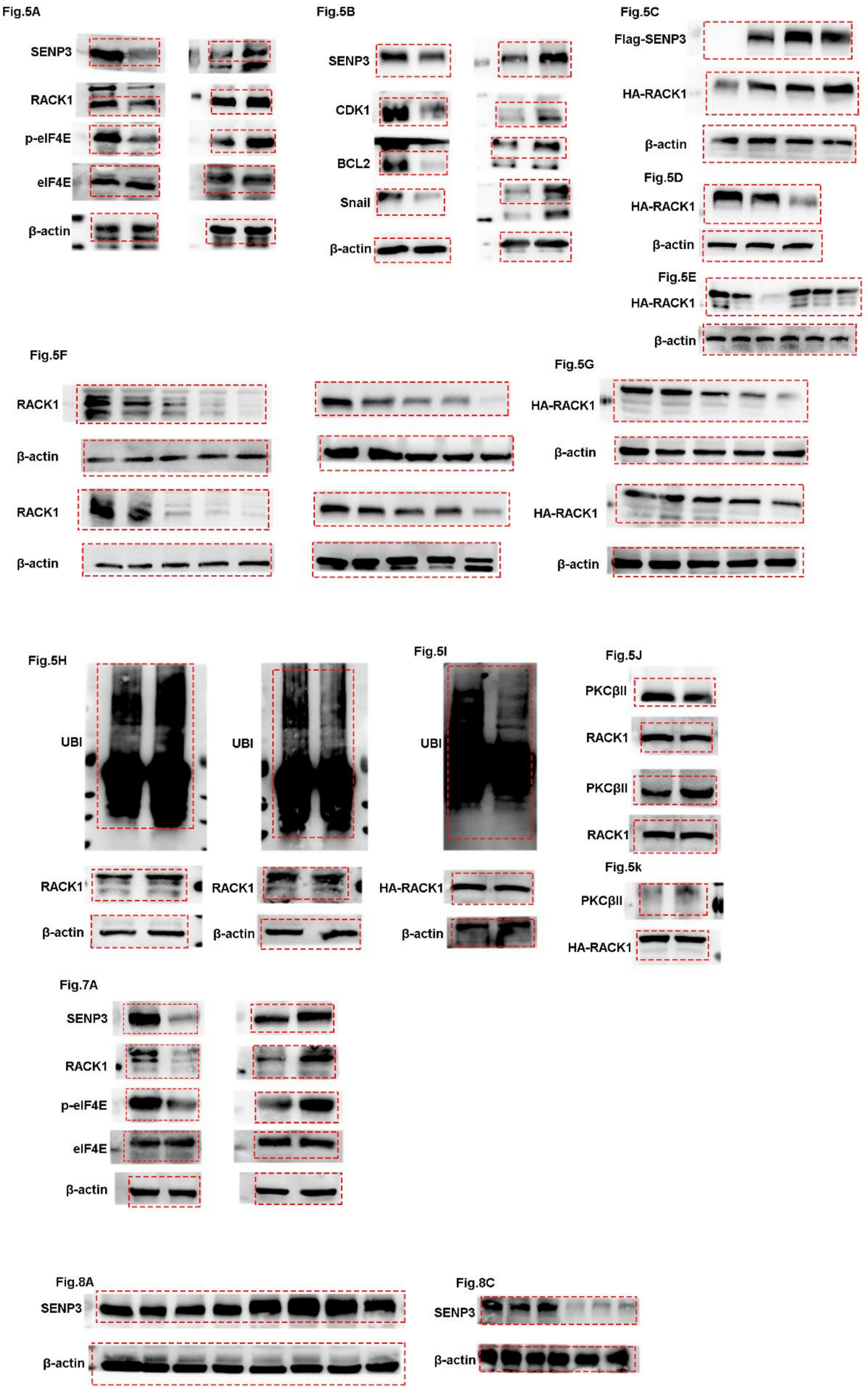
Fig.5A
SENP3
RACK1
p-eIF4E
eIF4E
β-actin
Fig.5B
SENP3
CDK1
BCL2
Snail
β-actin
Fig.5C
Flag-SENP3
HA-RACK1
β-actin
Fig.5D
HA-RACK1
β-actin
Fig.5E
HA-RACK1
β-actin
Fig.5F
RACK1
β-actin
RACK1
β-actin
Fig.5G
HA-RACK1
β-actin
HA-RACK1
β-actin
Fig.5H
UBI
UBI
RACK1
β-actin
RACK1
β-actin
Fig.5I
UBI
HA-RACK1
β-actin
Fig.5J
PKCβII
RACK1
PKCβII
RACK1
Fig.5k
PKCβII
HA-RACK1
Fig.7A
SENP3
RACK1
p-eIF4E
eIF4E
β-actin
Fig.8A
SENP3
β-actin
Fig.8C
SENP3
β-actin

Fig.S4A

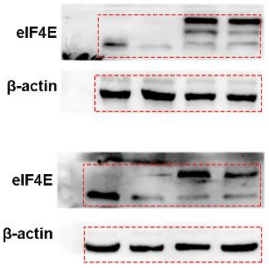

Fig.S5A

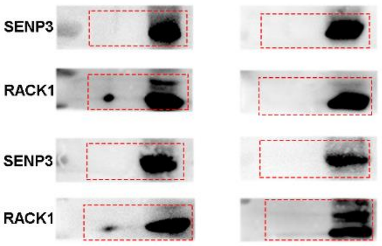

Fig.S5B

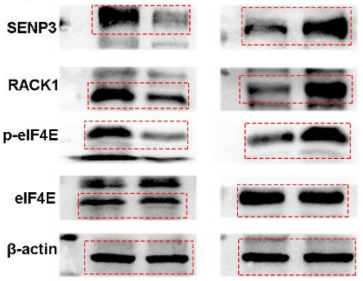

Fig.S5C

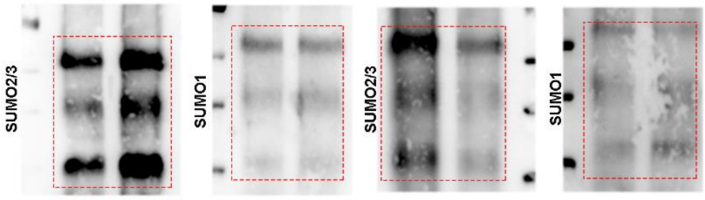

Fig.S5D

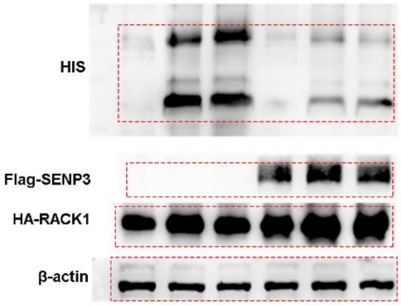

Fig.S5E

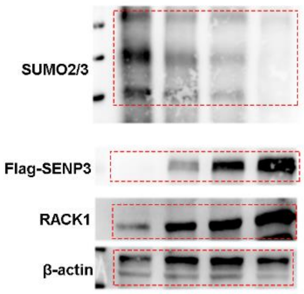

Fig.S5F

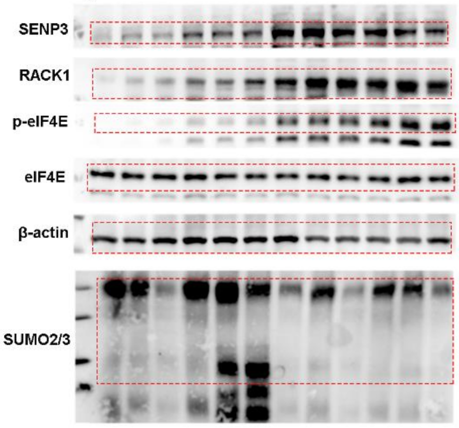

Fig.S5G

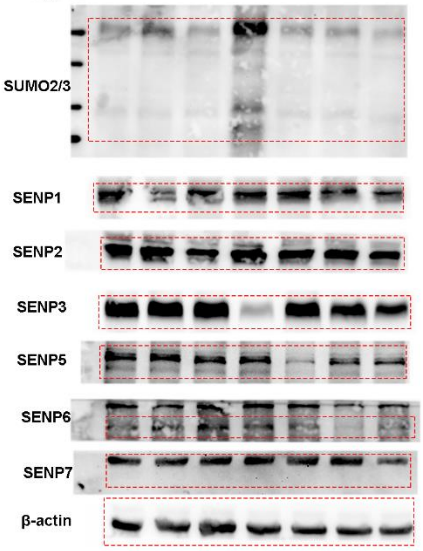

Fig.S6A

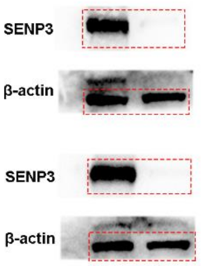

Fig.S6F

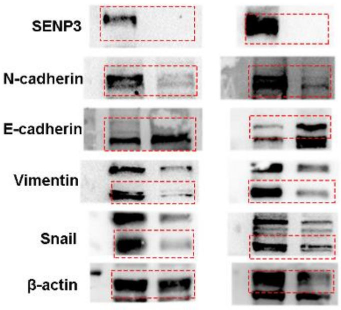

Supplement: Supplementary file 10 — Uncropped original western blots [file 41418_2024_1437_MOESM10_ESM.pdf]
